# Chromosome-level genome assembly of the sacoglossan sea slug *Elysia timida* (Risso, 1818)

**DOI:** 10.1186/s12864-024-10829-7

**Published:** 2024-10-07

**Authors:** Lisa Männer, Tilman Schell, Julia Spies, Carles Galià-Camps, Damian Baranski, Alexander Ben Hamadou, Charlotte Gerheim, Kornelia Neveling, Eric J. N. Helfrich, Carola Greve

**Affiliations:** 1 LOEWE Centre for Translational Biodiversity Genomics, Senckenberganlage 25, Frankfurt, 60325 Germany; 2 Senckenberg Research Institute, Senckenberganlage 25, Frankfurt, 60325 Germany; 3 Institute for Molecular Bio Science, Goethe University Frankfurt, Max-Von-Laue Straße 9, Frankfurt am Main, 60438 Germany; 4 Centre d’Estudis Avançats de Blanes (CEAB, CSIC), Accés Cala St. Francesc 14, Blanes, Girona, 17300 Spain; 5 Institut de Recerca de La Biodiversitat (IRBio), Universitat de Barcelona, Barcelona, Spain; 6 Department of Human Genetics, Radboud University Medical Centre (Radboudumc), Nijmegen, Netherlands

**Keywords:** High-quality reference genome, Arima HiC, PacBio HiFi, *Elysia timida*, Sacoglossa, Mollusca, Kleptoplasty, Polyketides, Biosynthesis

## Abstract

**Background:**

Sequencing and annotating genomes of non-model organisms helps to understand genome architecture, the genetic processes underlying species traits, and how these genes have evolved in closely-related taxa, among many other biological processes. However, many metazoan groups, such as the extremely diverse molluscs, are still underrepresented in the number of sequenced and annotated genomes. Although sequencing techniques have recently improved in quality and quantity, molluscs are still neglected due to difficulties in applying standardized protocols for obtaining genomic data.

**Results:**

In this study, we present the chromosome-level genome assembly and annotation of the sacoglossan sea slug species *Elysia timida*, known for its ability to store the chloroplasts of its food algae. In particular, by optimizing the long-read and chromosome conformation capture library preparations, the genome assembly was performed using PacBio HiFi and Arima HiC data. The scaffold and contig N50s, at 41.8 Mb and 1.92 Mb, respectively, are approximately 30-fold and fourfold higher compared to other published sacoglossan genome assemblies. Structural annotation resulted in 19,904 protein-coding genes, which are more contiguous and complete compared to publicly available annotations of Sacoglossa with respect to metazoan BUSCOs. We found no evidence for horizontal gene transfer (HGT), i.e. no photosynthetic genes encoded in the sacoglossan nucleus genome. However, we detected genes encoding polyketide synthases in *E. timida*, indicating that polypropionates are produced. HPLC–MS/MS analysis confirmed the presence of a large number of polypropionates, including known and yet uncharacterised compounds.

**Conclusions:**

We can show that our methodological approach helps to obtain a high-quality genome assembly even for a "difficult-to-sequence" organism, which may facilitate genome sequencing in molluscs. This will enable a better understanding of complex biological processes in molluscs, such as functional kleptoplasty in Sacoglossa, by significantly improving the quality of genome assemblies and annotations.

**Supplementary Information:**

The online version contains supplementary material available at 10.1186/s12864-024-10829-7.

## Introduction

Studying genomes of species is essential to comprehend the biology of organisms [1]. Third generation sequencing technologies, such as PacBio HiFi or Oxford Nanopore sequencing, have opened up the possibility of rapidly sequencing high-quality reference genomes of different organism groups at a reasonable price. However, sequencing methods and protocols are mainly developed and optimized for model organisms, especially human samples. In addition, DNA isolation for many non-model organisms is challenging. Sometimes even well-established sequencing methods sometimes do not work as expected, requiring special and adjusted handling [2]. Because of the existing bias towards developing methods for model species, certain taxonomic groups are still severely underrepresented in terms of genomic data and high-quality genome assemblies [3, 4].

Molluscs represent the second-largest animal phylum consisting of approximately 200,000 species – many of them still undescribed [5–7]. The diversity in molluscs is not only reflected in their manifold appearances, but also in divergent life cycles and habitats. Furthermore, they are of great ecological, economic, and medical significance [8, 9]. Considering their ecological and economical importance as well as species richness of the phylum, genomic resources of molluscs are still disproportionately low. It is worth noting that the number of molluscan reference genome assemblies on NCBI has more than doubled in recent years [10]. However, more high-quality and less fragmented genomes need to be sequenced and annotated to gain a deeper insight into the genomic diversity and evolutionary characteristics of molluscs.

**Fig. 1:**
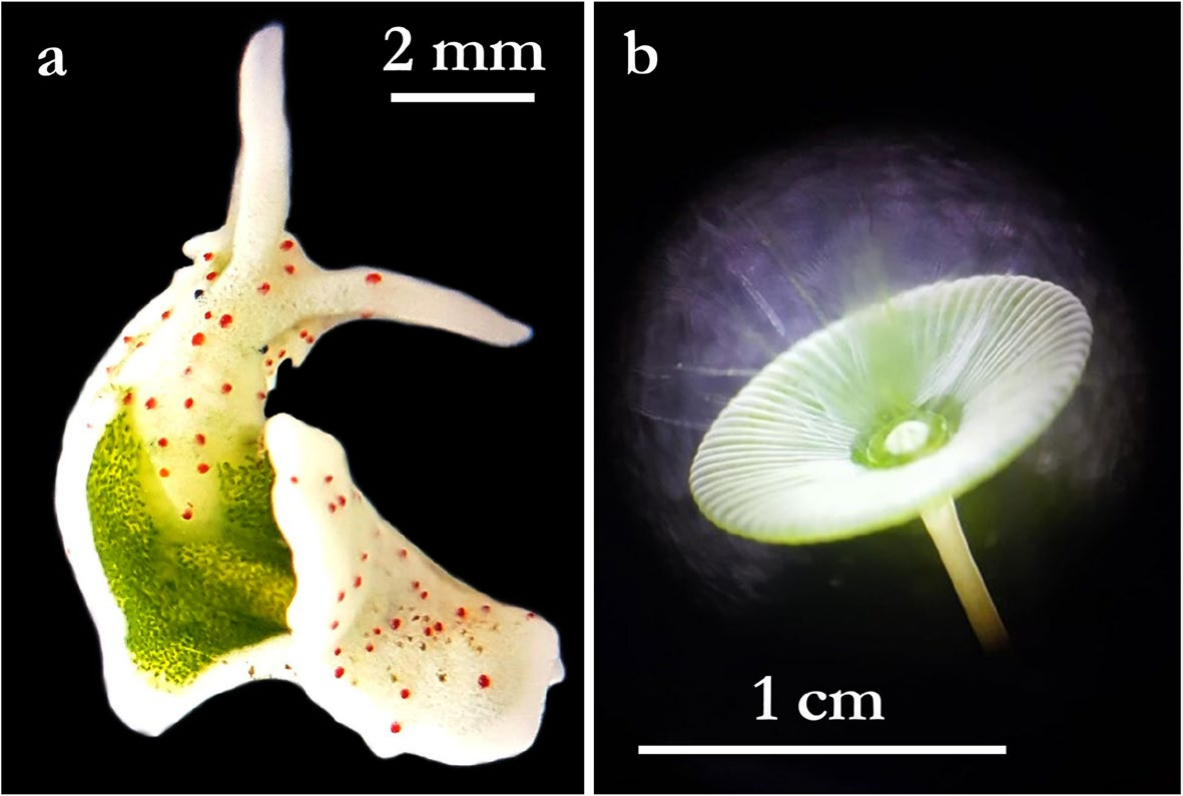
The Sacoglossa *Elysia timida* [24] (**a**) and its unicellular food algae *Acetabularia acetabulum* (**b**). Photos were taken by C. Greve

An extraordinary group within molluscs are the sacoglossans, also known as “solar-powered” sea slugs. Some sacoglossan species have evolved an exceptional photosynthetic association with chloroplasts and are able to functionally store them from their food algae in the cells of their digestive gland, a process which is also referred to as functional kleptoplasty [11–13]. The role of the incorporated functional chloroplasts in the nutrition and metabolism of sacoglossan sea slugs is still highly controversial among scientists [14–18]. Most sacoglossans digest the kleptoplasts immediately (non-retention types) or after a few weeks (short-term retention types). However, six sacoglossan species are known to be capable of long-term chloroplast retention, in which the incorporated chloroplasts remain photosynthetically active for a period of two to ten months [19–23], such as the shallow-water Mediterranean species *E. timida* [24–26] (Fig. 1a; https://youtu.be/MZRep08-81Y).

It still remains unknown how these sacoglossan species keep the chloroplasts active in their digestive gland cells without support from the algal nucleus, since chloroplasts need to import many proteins encoded by nuclear genes for their activity [27]. Previous research suggested horizontal gene transfer (HGT) of photosynthetic genes from the algal nuclear genome to the nuclear DNA of the sacoglossan sea slug [28–30], but this was not supported by subsequent studies [31–33]. To determine whether the nuclear genome of *E. timida* contains traces of HGT from algae, the assembled contigs were screened for algal-like sequences. Polypropionate pyrones, produced in sacoglossans, have been suggested to be involved in the establishment and the maintenance of the association between sacoglossa and the incorporated chloroplasts by serving antioxidant and photoprotective roles [34]. Therefore, to gain a better insight into the evolution and functionality of certain gene families, such as those encoding the polyketide synthases (PKS) involved in polypropionate pyrone biosynthesis, high-quality genome assemblies of Sacoglossa species are required in terms of completeness and contiguity.

In this work, we present the first annotated chromosome-level genome assembly of *E. timida*, and compare it with publicly available sacoglossan genomes. In addition, we provide a laboratory protocol that can be used to improve the sequencing of DNA from organisms whose sequencing is severely hampered by, for example, precipitated contaminants and DNA-bound metabolites that inhibit the sequencing polymerase. However, sequencing of such organisms can be improved by amplification-based protocols using currently available technologies and library kits [35]. This newly generated high-quality genome assembly may serve as a reference genome for future genetic investigations on kleptoplasty in *E. timida* and as a high-quality resource for studies on sacoglossans and molluscs in general.

## Material & methods

### Sample collection and sequencing

Specimens of *E. timida* (Fig. 1a) were collected in Cadaqués, Girona, Spain, in June 2021 (coordinates: 42.285173, 3.296461). Genomic DNA was extracted from a single individual using a CTAB-based method [36]. First, we prepared two PacBio ultra-low input libraries (including a long-range PCR amplification step using the PacBio polymerases A/B) using the SMRTbell® gDNA Sample Amplification Kit and the SMRTbell® Express Template Preparation Kit 2.0. Two SMRT cell sequencing runs were performed on the Sequel System IIe in CCS mode. In addition, to reduce potential PCR biases of the amplification polymerases A/B supplied by PacBio, we prepared two further libraries using the KOD Xtreme™ Hot Start DNA Polymerase (Merck), optimized for amplification of long and GC-rich DNA templates. For this, we combined the buffer, dNTPs and KOD polymerase from the KOD Xtreme Hot Start DNA Polymerase Kit with the ultra-low input primers from the PacBio SMRTbell gDNA Sample Amplification Kit. Otherwise, we followed the PacBio ultra-low input protocol (see also protocol adaptation and cycling conditions in Supplemental Tables S5). To obtain sufficient DNA to generate the SMRTbell libraries with the customized PacBio ultra-low input protocol, we performed two independent amplifications with the KOD polymerase and then pooled the DNA. These two customized PacBio ultra-low input libraries were then each sequenced on a single SMRTcell using the PacBio Sequel IIe and Revio instruments, respectively. An initial attempt to sequence a PacBio standard/low-input library of these animals resulted in very poor sequencing results (Supplemental Table S6). The same was true for the attempt to sequence *E. timida* with Oxford Nanopore technology.

Chromatin conformation capture libraries were prepared using the Arima HiC Kit v01 (Arima Genomics) according to the manufacturer's low-input protocol with a slight modification in the initial sample preparation steps. Another whole specimen of *E. timida* from the same locality was first washed in seawater, then in deionised water, and finally ground with a pestle in a 1.5 mL tube. After preparing the specimen, we followed the manufacturer's instructions for proximity ligation. The proximally-ligated DNA was then converted into an Arima High Coverage HiC library according to the protocol of the Swift Biosciences® Accel-NGS® 2S Plus DNA Library Kit. The fragment size distribution and concentration of the Arima High Coverage HiC library was assessed using the TapeStation 2200 (Agilent Technologies) and the Qubit Fluorometer and Qubit dsDNA HS reagents Assay kit (Thermo Fisher Scientific, Waltham, MA), respectively. The library was sequenced on the NovaSeq 6000 platform at Novogene (UK) using a 150 paired-end sequencing strategy, with an expected output of 30 Gb.

RNA was extracted from a third individual from the same locality using TRIzol reagent (Invitrogen) according to the manufacturer's instructions. Quality and concentration were assessed using the TapeStation 2200 (Agilent Technologies) and the Qubit Fluorometer with the RNA BR Reagents Assay Kit (Thermo Fisher Scientific, Waltham, MA). The RNA extraction was then sent to Novogene (UK) for Illumina paired-end 150 bp RNA-seq of a cDNA library (insert size: 350 bp) sequenced on a NovaSeq 6000, with an expected output of 12 Gb.

### Genome size estimation

The genome size was estimated following a flow cytometry (FCM) protocol with propidium iodide-stained nuclei described by Hare and Johnston (2012) [37]. Two fresh individuals of *E. timida* from Cadaqués were homogenized in a 1.5 mL tube with a pestle, and, as an internal reference standard, neural tissue from *Acheta domesticus* (female, 1C = 2 Gb) was chopped with a razor blade in a petri dish. Ice-cold Galbraith buffer (2 mL) was used as the suspension medium. The suspensions were filtered each through a 42-μm nylon mesh, then stained with the intercalating fluorochrome propidium iodide (PI, Thermo Fisher Scientific) (final concentration 25 µg/mL), and treated with RNase A (Sigma-Aldrich) (final concentration 250 µg/mL). The mean red PI fluorescence signal of stained nuclei was quantified using a Beckman-Coulter CytoFLEX flow cytometer with a solid-state laser emitting at 488 nm. Fluorescence intensities of at least 10,000 nuclei per measurement were recorded. We used the software CytExpert 2.3 for histogram analyses. After measuring the suspensions of *E. timida* and the internal reference standard separately, they were mixed. With this suspension mix, the total quantity of DNA per nuclei of *E. timida* was calculated as the ratio of the mean red fluorescence signal of the 2C peak of the stained nuclei of *E. timida* divided by the mean fluorescence signal of the 2C peak of the stained nuclei of the reference standard times the 1C amount of DNA in the reference standard. In total, two suspensions from two *E. timida* individuals were measured, each with four replicates that were measured on four different days to minimize possible random instrumental errors. The average of these eight measurements was calculated to estimate the genome size (1C) of *E. timida*. The value of the robust coefficient of variance (rCV), which should be about 5% or less, provides an estimate of the confidence level of the measurements.

Genome size and heterozygosity were estimated from a k-mer profile of the HiFi reads. First, count from Jellyfish 2.3.0 [38] was run with the additional parameters “-F 4 -C -m 21 -s 1,000,000,000 -t 96” and all HiFi reads as input. Second, a histogram was created from the resulting database with “jellyfish histo -t 96”. Third, GenomeScope 2.0 [39] in combination with R 4.3.1 was executed using the histogram as input. Additionally, *E. timida*´*s* genome size was also estimated from coverage distribution of mapped PacBio reads using ModEst [40], as implemented in backmap 0.5 [3] (https://github.com/schellt/backmap).

### Assembly strategy

Bioinformatic analyses were conducted with default parameters if not stated otherwise.

HiFi reads were called using a pipeline, which is running PacBio’s tools ccs 6.4.0 (https://github.com/PacificBiosciences/ccs), actc 0.3.1 (https://github.com/PacificBiosciences/actc), samtools 1.15 [41], and DeepConsensus 1.2.0 [42]. All commands were executed as recommended in the respective guide for DeepConsensus (https://github.com/google/deepconsensus/blob/v1.2.0/docs/quick_start.md) except --all was applied instead of --min-rq = 0.88 for ccs.

To remove PCR adapters and PCR duplicates, which might originate from the PCR amplification during the ultra-low library preparation, PacBio’s tools lima 2.6.0 (https://github.com/PacificBiosciences/barcoding) with options “--num-threads 67 --split-bam-named --same --ccs” and pbmarkdup 1.0.2–0 with options “--num-threads 84 --log-level INFO --log-file pbmarkdup.log --cross-library --rmdup” (https://github.com/PacificBiosciences/pbmarkdup) were applied, respectively.

We assembled the genome of *E. timida* from filtered PacBio HiFi reads of four SMRT cells using hifiasm 0.19.8 [43]. Subsequently, the primary contigs were processed. Contamination was filtered out by first running “screen genome” of FCS-GX 0.5.0 [44] with the corresponding database (downloaded on Dec 5th, 2023) and the NCBI taxonomy ID (154,625). Second, “clean genome” was executed with the action report created by “screen genome” and a minimum sequence length of 1 (--min-seq-len 1). Subsequently, the FCS filtered assembly was polished using a workflow which includes DeepVariant. First, the HiFi reads used for assembly were mapped against the contigs with minimap2 2.26 [45, 46] and the options “-a -x map-hifi”. The bam file was sorted by coordinate with samtools 1.19.1 and duplicated HiFi reads were removed with Picard 3.1.0 [47] MarkDuplicates and the option --REMOVE_DUPLICATES. The assembly fasta and filtered bam files were indexed with samtools faidx and index commands, respectively. To call SNPs, DeepVariant 1.5.0 [48] was applied. To keep only homozygous variants, SNPs were subsequently filtered using bcftools view 1.13 [41] with the options -f 'PASS' -i 'GT = "1/1"'. Then, the vcf file containing the homozygous SNPs was indexed with tabix from htslib 1.17 [49] to finally apply the variants in the filtered assembly with bcftools consensus, which is from here on referred to as polished assembly. Haplotigs were purged from the polished assembly with purge_dups 1.2.6 (https://github.com/dfguan/purge_dups) together with minimap 2.24 for mapping HiFi reads and self-alignment of the assembly according to the guidelines (https://github.com/dfguan/purge_dups/tree/v1.2.6?tab=readme-ov-file#--pipeline-guide), except “-x map-hifi” was applied during HiFi mapping and high coverage contigs were kept when running get_seqs (-c). Prior to HiC scaffolding, blobtoolkit 4.1.4 [50] was used to evaluate if contamination was still present. Taxonomic assignment for blobtools was conducted with blastn 2.15.0+ [51] and the options “-task megablast -outfmt '6 qseqid staxids bitscore std' -num_threads 96 -evalue 1e-25”. Information on coverage per contig was obtained from mapping HiFi reads used for assembly back to the assembly itself via backmap 0.5 [3, 40] in combination with minimap 2.26 [45], samtools 1.17, Qualimap 2.3 (bamqc; [52]), bedtools 2.30.0 [53] and R 4.0.3 [54]. Contigs were excluded if taxonomic assignment was not one of no-hit, Mollusca, Chordata or Arthropoda, GC was lower than 0.287, or average coverage was lower than 15.

The polished contigs that were filtered via blobtools, were scaffolded with Arima HiC reads in yahs 1.1 [55]. To do so, HiC reads were first mapped with the Arima mapping pipeline (https://github.com/VGP/vgp-assembly/blob/master/pipeline/salsa/arima_mapping_pipeline.sh), in combination with bwa mem 0.7.17 [56], samtools 1.15.1, picard 2.27.1, and java 1.8.0 [57]. Afterwards, the bam file was processed along with the contigs in yahs. A hic file was created with the tool “juicer pre” from yahs, which was loaded together with the respective assembly file into Juicebox 1.11.08 [58, 59] for manual curation.

To test for support on HGT between photosynthetic genes from the algal nuclear genome to the nuclear DNA of *E. timida*, the blast search used for taxonomic assignment in blobtools was screened for hits of *Acetabularia acetabulum* and *Ulva compressa* outside the contigs assigned as Chlorophyta. Furthermore, Chlorophyta blast hits were screened if respective database sequences are chloroplast or nuclear sequences. Finally, a HiC scaffolding was conducted as explained above, except that all contigs, which were filtered out due to taxonomic assignment to Chlorophyta, were added again to the polished and blobtools filtered contig set.

Contigs of mitochondrial origin were filtered out after manual curation. To do so, MitoHiFi 3.2.1 [60] was applied together with the available mitochondrial genome sequence of *E. timida* (KU174946.1; [61]) and the scaffolds after manual curation. Subsequently, blastn 2.15.0+ was applied to find curated scaffolds similar to our *E. timida* mitochondrial genome sequence. All scaffolds with an alignment length equal to the mitogenome length were filtered out. Additionally, all contigs flagged as circular by hifiasm were filtered out, which only included sequences that remained un-scaffolded.

During different stages of the assembly, quality controls were conducted. Basic contiguity statistics were calculated with Quast 5.2.0 [62]. Single copy orthologs of the provided metazoan set were searched with BUSCO 5.5.0 [63]. Completeness regarding k-mers and QV values were obtained with Meryl 1.3 and Merqury 1.3 [64]. Mapping coverage distribution of HiFi reads was checked using backmap 0.5.

### Annotation

Masking of repetitive regions was conducted by identifying repeat families with RepeatModeler 2.0.5 [65, 66] and the dependencies rmblast 2.14.1+ as search engine, and TRF 4.09 [67], RECON 1.08 [68], RepeatScout 1.0.6 [69], as well as RepeatMasker 4.1.6 [70]. LTR structural discovery pipeline was enabled (-LTRStruct) with the dependencies GenomeTools 1.6.2 [71], LTR_Retriever 2.9.9 [72], Ninja 0.97 [73], MAFFT 7.520 [74], and CD-HIT 4.8.1 [75]. The resulting repeat families were used as repeat library in RepeatMasker 4.1.6 [70] together with the options “-xsmall -no_is -e ncbi -pa 10 -s” and the dependencies rmblast 2.14.1+ as search engine, HMMer 3.4 (hmmer.org) andTRF 4.09 [67].

Structural annotation of the *E. timida* genome assembly, was conducted with BRAKER 3.0.8 [76–91]. RNAseq data from *E. timida* was mapped against the genome assembly using HISAT 2.2.1 [92], and the bam file sorted by coordinate with samtools 1.19.1 was provided to BRAKER with --bam. In addition, we downloaded the protein sets from the high-quality annotations of six molluscan species, which were then used as evidence during structural annotation: *Aplysia californica* (GCF_000002075.1; [93]), *Gigantopelta aegis* (GCF_016097555.1; [94]), *Mizuhopecten yessoensis* (RefSeq: GCF_002113885.1; [95, 96]), *Octopus sinensis* (GCF_006345805.1; [97]), *Pecten maximus* (GCF_902652985.1; [98], and *Pomacea canaliculata* (RefSeq: GCF_003073045.1; [99, 100]) (Table 1). Before the protein sequences were concatenated and provided with --prot_seq to BRAKER, we evaluated BUSCO completeness, number of genes, and corresponding contiguity of the annotation to verify the high quality of the mollusc protein set. BRAKER was executed with the additional parameters --gff3 --threads=96 --busco_lineage=metazoa_odb10.

**Table 1:** The protein sets used as evidence for the annotation of the genome assembly

Species	Metazoa BUSCO (*n*: 954)	Number of protein sequences
*Aplysia californica*	C:97.8%[S:75.8%,D:22.0%],F:0.8%,M:1.4%	26,656
*Gigantopelta aegis*	C:98.5%[S:70.5%,D:28.0%],F:0.8%,M:0.7%	33,249
*Mizuhopecten yessoensis*	C:98.6%[S:75.2%,D:23.4%],F:0.4%,M:1.0%	41,567
*Octopus sinensis*	C:98.4%[S:69.2%,D:29.2%],F:0.8%,M:0.8%	36,112
*Pecten maximus*	C:98.5%[S:74.7%,D:23.8%],F:0.4%,M:1.1%	39,918
*Pomacea canaliculata*	C:98.2%[S:74.9%,D:23.3%],F:0.4%,M:1.4%	40,391

Quality controls of the *E. timida* annotation were conducted by searching for single copy orthologs with BUSCO and by calculating basic contiguity statistics of the annotated features (e.g. genes, mRNAs, etc.). The functional annotation of the predicted *E. timida* proteins was conducted using InterProScan 5.64–96.0 [101]. Databases and tools which were used during the operation with InterProScan [102], are shown in detail in Supplemental Table S1. Furthermore, InterProScan was executed in the same way using the four protein sets from annotations of *Elysia chlorotica*, *Elysia crispata*, *Elysia marginata,* and *Plakobranchus ocellatus* as input (Table 4).

### Comparison of PKS encoding genes from sacoglossans

To detect the presence and copy number of PKS genes in the annotation of *E. timida,* the PKS coding sequences from the genomes of *E. chlorotica*, *Elysia diomedea* and *P. ocellatus* from Torres et al. (2020) [34] were used as a reference (Supplemental Table S2). We also evaluated the presence and copy number of PKS genes in the annotations of *E. chlorotica*, *E. crispata*, *E. marginata* and *P. ocellatus*.

Nucleotide sequences were translated into their corresponding six protein reading frames using Geneious Prime 2023.0.4. We proceeded with the reading frames which did not contain any stop codon. These sequences were then blasted against the annotations of *E. timida*, *E. chlorotica*, *E. crispata, E. marginata*, and *P. ocellatus* using blastp 2.14.0+ [51] and the options “-task blastp -outfmt '6 qseqid staxids bitscore std qlen slen' -evalue 1e-25”. We furthermore filtered out all blast hits with an identity lower than 80%.

### Phylogenetic analysis of FASs and PKSs

Transcript sequences of the FASs and PKSs from *E. timida*, *E. chlorotica*, *E. diomedea* and *P. ocellatus* were retrieved, MUSCLE aligned, manually refined and realigned with MUSCLE. A maximum likelihood phylogenetic tree was created in MEGA11 using 1000 bootstrap replications [103]. The branch lengths are scaled according to the number of substitutions per site.

### Breeding conditions of *E. timida* for polypropionate extraction

Individuals of *E. timida* from Cadaqués were reared in a climate chamber at 20 °C, where they spawned. Hatchling specimens (F1) were reared to adulthood under the same conditions as their parental line. The sea slugs were housed in small and transparent plastic containers and kept in a 12:12 day-night rhythm (153 lx; λp: 545 nm) (Supplemental Figure S1). Once a week water was changed and food algae (*A. acetabulum*; Fig. 1b) were provided.

### Extraction and HR-HPLC–MS measurement

Three F1 specimens were independently homogenized by blending in 0,2 Mol Tris–HCl at pH 7 in a volume of 1 mL at room temperature. The extraction was performed using ethyl acetate in tenfold excess. The crude extract was dried under reduced pressure and re-dissolved in methanol. The extracts were measured by high-performance liquid chromatography-electrospray ionization high-resolution mass spectrometry (HPLC-ESI-HR-MS) using an Ultimate 3000 LC system coupled to an ImpactII QTOF (Bruker) high-resolution mass spectrometer. The extract was separated on an Acquity UPLC BEH C18 column (130 Å, 1.7 µm particle size, 2.1 mm × 100 mm) with a gradient flow of 0,4 mL/min from 5 to 95% solvent B (acetonitrile + 0,1% formic acid) over a time span of 14 min. The data was acquired in positive mode at a scan range between *m/z* 100 to *m/z* 1200 and analyzed using the Bruker software DataAnalysis 4.3 and MetaboliteDetect 2.1. HPLC was used to separate the constituents of the crude extract based on their physico chemical properties, high resolution mass spectrometry was used to determine the exact mass and sum formula of the compounds of interest and tandem mass spectrometry results in characteristic fragmentation patterns (fingerprints) of each molecule. The MS/MS fingerprints of characterized polypropionates were used to identify related compounds based on their similar MS/MS fragmentation patterns to characterized polypropionates as reported by Torres et al. (2020) [34]. Similar MS/MS fragmentation patterns are indicative of structural relatedness between two compounds.

### Molecular networking and visualization

The HPLC–MS/MS datasets obtained from analysis of the crude extracts from *E. timida* specimens were uploaded to Global Natural Products Molecular Networking (GNPS) to generate a molecular network, setting the minimum matched peaks to 7 and the cosine score to 0.6 [104]. The software Cytoscape 3.9.1 was used to visualize the molecular network [105]. The excerpt of the network that visualizes the polypropionates was identified by the presence of nodes representing characterized compounds [34]. Putative polypropionates were identified based on their exact mass (sum formula) and similar fragmentation patterns to characterized polypropionates.

### Construction of proposed sequence for EtPKS1 mRNA

The putative sequence of the mRNA for *E. timida* PKS1 (EtPKS1) was proposed based on sequence similarity with the transcript for the EcPKS1 (Accession number: MT348433). The conserved “GHSMGE” motif in the acyltransferase domain of PKS1 was identified on nucleotide level and used as a bait for the identification of the genomic area encoding the EtPKS1 [34]. An excerpt of the genomic sequence around this sequence motif was translated in the three forward translation frames to map the translations with the EtPKS1 transcript and annotate the exons.

## Results

### Genome size estimation

Flow cytometry results were represented as histograms displaying the relative propidium iodide fluorescence intensity which we received after a simultaneous analysis of *E*. *timida* 2C and the house cricket *A. domesticus* 2C as an internal reference standard (Supplemental Figure S2). The obtained average haploid genome sizes for the two individuals of *E. timida* were 898.00 and 891.78 Mb, respectively. From all eight measurements, including both individuals, a genome size of 894.89 Mb was estimated (Supplemental Table S3).

Mapping based genome size estimation with ModEst based on the final genome assembly resulted in 632 Mb. Based on k-mers, the genome size was estimated to be 548.2 Mb and heterozygosity 0.794% (Supplemental Figure S3). The heterozygosity value of *E. timida* is higher compared to other sacoglossan species (Supplemental Table S4).

### Sequencing

The four PacBio sequencing runs using the standard PacBio ultra-low and the customized PacBio ultra-low DNA input library preparations yielded a total polymerase read length of 510 Gb, 431 Gb, 612 Gb, and 1,360 Gb, respectively (Supplemental Table S6 and Supplemental Figure S4). Illumina sequencing of Arima HiC and RNAseq libraries resulted in 95.7 and 40.6 million read pairs, corresponding to 28.7 and 12.2 Gb, respectively.

Raw data of PacBio HiFi reads (subreads) and raw Arima HiC reads which were used for genome assembly, raw RNAseq reads as well as the final assembly and annotation can be publicly accessed via BioProject PRJNA1119176 and this link: https://genome.senckenberg.de/download/etim/.

### Assembly

**Fig. 2:**
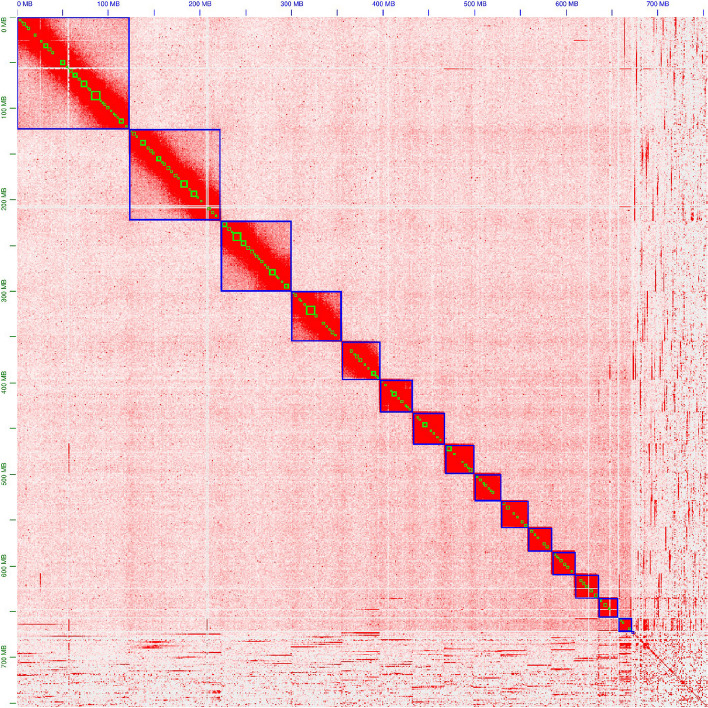
Contact map after yahs scaffolding using Arima HiC data and manual curation. Blue and green squares mark scaffolds and contigs, respectively. Higher number of contacts is represented by higher intensity of the colour

**Fig. 3:**
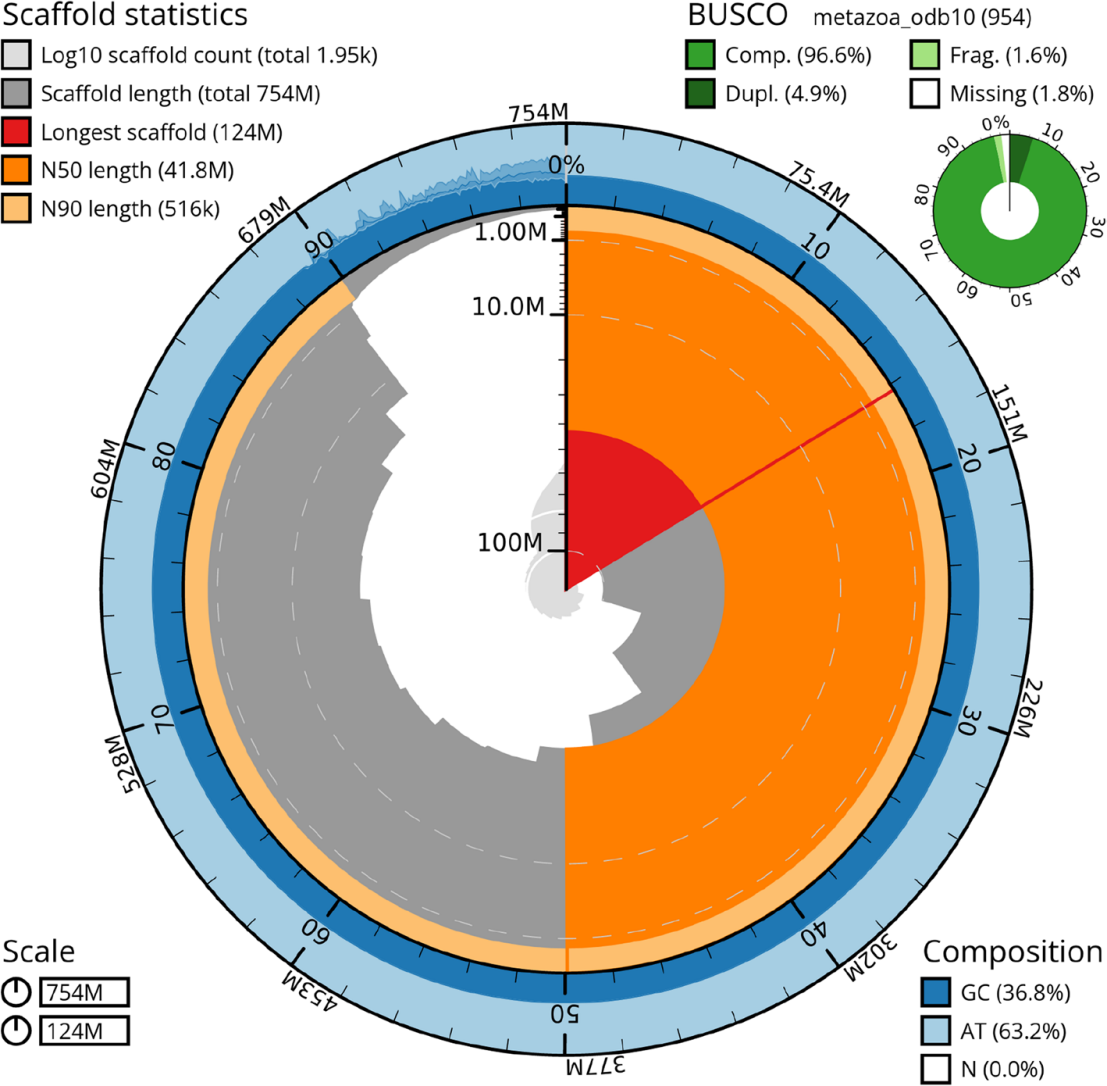
Snail plot of the final genome assembly. The plot created with blobtoolkit visualizes amongst others scaffold count, lengths, length distribution, nucleotide composition, and recovered BUSCOs

HiFi calling and subsequent PCR duplicate removal resulted in more than 19 million HiFi reads with a total length of 108 Gb and an N50 of 6,143 bp (more details in Supplemental Figure S4). Given the genome size estimates based on FCM (895 Mb) and ModEst (632 Mb), the theoretical coverages were calculated to be 120x and 170x, respectively. FCS-GX identified 873 sequences (53.3 Mb) containing contamination (Supplemental Table S7), which were excluded or trimmed (Supplemental Table S8). Nevertheless, after polishing and purging, the blobplot still showed the presence of contamination (Supplemental Figure S5). Blobtools returned five sequences taxonomically assigned to Chlorophyta, of which three were assigned to *A. acetabulum* and two to *U. compressa* (Supplemental Table S9). In total, 3084 additional contigs (100.8 Mb), including the five assigned to Chlorophyta, were removed before proceeding with HiC scaffolding (blobplot after removal of contamination in Supplemental Figure S11). After manual curation, scaffolding resulted in 15 chromosome-scale sequences (Fig. 2), showing no contamination (Supplemental Figure S6). These 15 scaffolds made up 89.1% of the assembly’s total length. The total length of the final *E. timida* genome assembly was 754 Mb with a scaffold and contig N50 of 41.8 Mb and 1.92 Mb, respectively. Furthermore, the assembly had a QV of 58.3. Additional quality metrics of the final *E. timida* assembly and a comparison to publicly available sacoglossan genome assemblies are shown in Fig. 3 and Table 2.

**Table 2:** Quality metrics of the *E. timida* genome assembly in comparison to available sacoglossan species

	***Elysia timida***	***Elysia chlorotica***	***Elysia marginata***	***Plakobranchus ocellatus***	***Elysia crispata***
Citation	This study	[106]	[33]	[33]	[107]
Accession number		GCA_003991915.1	GCA_019649035.1	GCA_019648995.1	GCA_033675545.1
**Contigs**					
** Number of sequences**	2539	41,624	211,550	280,271	8089
** Total length (Mb)**	754.4	540.5	707.4	849.1	786.4
** N50 (Mb)**	1.92	0.03	0.006	0.005	0.45
**Scaffolds**					
** Number of sequences**	1946	9987	14,149	8647	-
** Total length (Mb)**	754.5	557.5	790.3	927.9	-
** N50 (Mb)**	41.8	0.44	0.23	1.45	-
** % of Ns**	0.016	3.04	10.43	8.48	-
**BUSCO (%; *N* = 954)**					
** C**	96.6	94.8	89.2	95.3	96.9
** S**	91.7	94.1	88.5	94.7	96.3
** D**	4.9	0.7	0.7	0.6	0.6
** F**	1.6	3.1	7.2	2.7	1.6
** M**	1.8	2.1	3.6	2.0	1.5

The contig N50 of the *E. timida* genome assembly is fourfold higher than the highest contig N50 so far achieved for a sacoglossan species’ genome assembly (*E. crispata*: 0.45 Mb), while the BUSCO values of *E. timida* were similar to those of the other sacoglossans (Table 2). Only duplicated BUSCOs were higher for *E. timida* compared to the other assemblies. As HiC data have not yet been generated for other Sacoglossa, the *E. timida* assembly has a manyfold higher scaffold N50 in comparison (Table 2).

### Checking for support regarding horizontal gene transfer

The sequence similarity search for taxonomic assignment in blobtools returned five contigs, which were classified as *A. acetabulum* or *U. compressa* (see assembly results). Further inspection of the blast output showed that the five Chlorophyta contigs only generated hits leading to taxids of *A. acetabulum* (NCBI:txid35845) or *U. compressa* (NCBI:txid63659) and not to any *E. timida* contigs (Supplemental Table S10). All target sequences of blast hits with taxids of *A. acetabulum* or *U. compressa* are sequences from a chloroplast of these two species (Supplemental Table S11). HiC scaffolding was performed to test whether the five Chlorophyta contigs could be linked to other nuclear sequences of *E. timida*. This resulted in splitting one contig but none of the Chlorophyta contigs was linked to other *E. timida* sequences (Supplemental Table S12).

### Annotation

RepeatModeler identified sequences of 1,886 repeat families with a total length of 1,705,165 bp. Subsequently, RepeatMasker annotated 44.3% of the assembly as repetitive, of which the majority of repetitive families were labelled as unclassified (32.5% of the assembly). Contiguity statistics and BUSCO results of the *E. timida* annotation compared to other Sacoglossa annotations are shown in Table 3. The values of the complete BUSCOs range between 86.1% and 92.9% which confirm the high quality of the protein sets of the published sacoglossan annotations.

**Table 3:** Quality metrics of the *E. timida* genome annotation in comparison to available sacoglossan species. *For annotations of *E. marginata* and *P. ocellatus* only “gene” and “CDS” features are annotated, so that no metrics regarding mRNAs and mean CDSs/mRNA as well as single CDS genes are shown

	***Elysia timida***	***Elysia chlorotica***	***Elysia marginata***	***Plakobranchus ocellatus***	***Elysia crispata***
Number					
Gene	15,809	23,871	70,752	77,230	67,429
mRNA	19,904	23,871	-	-	70,120
CDS	211,587	155,011	249,028	271,856	329,620
Mean ratio					
mRNAs/ gene	1.26	1.00	-	-	1.04
CDSs/ mRNA	10.63	6.49	3.52*	3.52*	4.70
Median length (bp)					
Gene	9,325	5,452	1,708	1,561	2,419
mRNA	10,579	5,452	-	-	2,514
CDS	126	132	147	148	134
Total space (bp)					
Gene	236,595,571	215,824,722	314,620,341	363,188,163	375,920,318
mRNA	236,595,571	215,824,722	-	-	375,682,798
CDS	28,112,659	30,877,231	55,967,689	61,040,586	64,065,507
Number of single					
Exon mRNAs	1,441	3,514	-	-	16,366
CDS mRNAs	1,441	3,514	23,332*	28,380*	16,366
BUSCO (%; *N* = 954)					
C	98.7	91.5	86.1	91.1	92.9
S	75.4	91.2	85.3	89.9	87.2
D	23.3	0.3	0.8	1.2	5.7
F	0.4	3.8	9.1	6.2	2.7
M	0.9	4.7	4.8	2.7	4.4

In comparison to annotations of other sacoglossan genome annotations, *E. timida* was the most complete and least fragmented in terms of BUSCOs. Additionally, mean CDS per mRNA and median gene length among others were also highest for *E. timida*, while the number of single CDS mRNAs was lowest. Total gene and CDS space were similar or lower compared to other annotated sacoglossan species.

InterProScan assigned at least one functional annotation to 19,489 (97.9%) different *E. timida* protein sequences with one of the applied analyses. Furthermore, 12,368 (62.1%) different *E. timida* protein sequences were annotated with at least one Gene Ontology (GO) term, whereas this percentage was lower in other Sacoglossa with a wider range (from 22 to 46%). A comparison of functionally annotated protein sequences with other Sacoglossa can be found in Table 4.

**Table 4:** Number of protein sequences annotated structurally (Total) and functionally with at least one analysis in InterProScan or with at least one GO term

	***Elysia timida***	***Elysia chlorotica***	***Elysia marginata***	***Plakobranchus ocellatus***	***Elysia crispata***
Total	19,904	23,871	70,752	77,230	70,120
Functional annotated	19,489 (97.9%)	21,889 (91.7%)	61,319 (86.7%)	59,564 (77.1%)	46,665 (66.6%)
GO term annotation	12,368 (62.1%)	10,979 (46.0%)	18,150 (25.7%)	17,772 (23.0%)	15,626 (22.3%)

### PKS presence in *E. timida* and comparison of PKSs in sacoglossans

All genes encoding FAS and PKS from *E. chlorotica*, *E. diomedea* and *P. ocellatus* [34] were found in the annotation of *E. timida* using BLAST search. The FAS and PKS protein sets were also blasted against the genome annotations of *E. chlorotica*, *E. crispata*, *E. marginata* and *P. ocellatus* and found in all of them (Supplemental Figure S7 and Supplemental Table S13). The transcripts of the genes associated with FASs form a distinct clade from the PKS transcripts. The PKS1 transcripts and PKS2 transcripts also form two separate clades. The transcripts of *P. ocellatus* were the most phylogenetically distinct sequences for FAS, PKS1 and PKS2 compared to the transcripts of the *Elysia* species. Before filtering the blast hits, the nine PKS or FAS encoding genes from three sacoglossan species were detected in all of the other sacoglossans. To correct for false positives, we filtered out all the blast hits with an e-value above 1e-25 and a percentage of identical positions below 80%. After filtering, the number of hits reduced drastically in all sacoglossans (Supplemental Table S13). Before filtering, between two and 38 gene hits were found in all sacoglossan species. However, after filtering, the maximum number of gene copies was found in the annotation of *E. marginata*, where we had four blast hits of the EcPKS1 gene. Some sacoglossan annotations had no FAS or PKS gene hits after filtering. The genes encoding EtFAS, EtPKS1 and EtPKS2 were annotated in the genome of *E. timida* (Table 5). The genes encoding the EtFAS and EtPKS2 were automatically annotated by BRAKER 3.0.8. In contrast, the gene encoding the EtPKS1 was not automatically recognized, and was manually annotated based on the alignment with the amino acid sequence of EcPKS1 (Supplemental Figure S8). The presence of PKS encoding genes in the genome of *E. timida* was investigated to obtain an indication of which polypropionates can be produced by the sea slug.

**Table 5:** Sequence identifiers for the nucleotide and amino acid sequences of EtFAS, EtPKS1 and EtPKS2

Coding region in genome assembly	Transcript level	Protein level
Scaffold_4:11,989,323–12,013,722	g10811.t1	EtFAS
Scaffold_6:1,080,882–1152789	Assembled manually	EtPKS1
Scaffold_12:1,113,801–1144318	g4188.t1	EtPKS2

### Identification of putative polypropionates

**Fig. 4:**
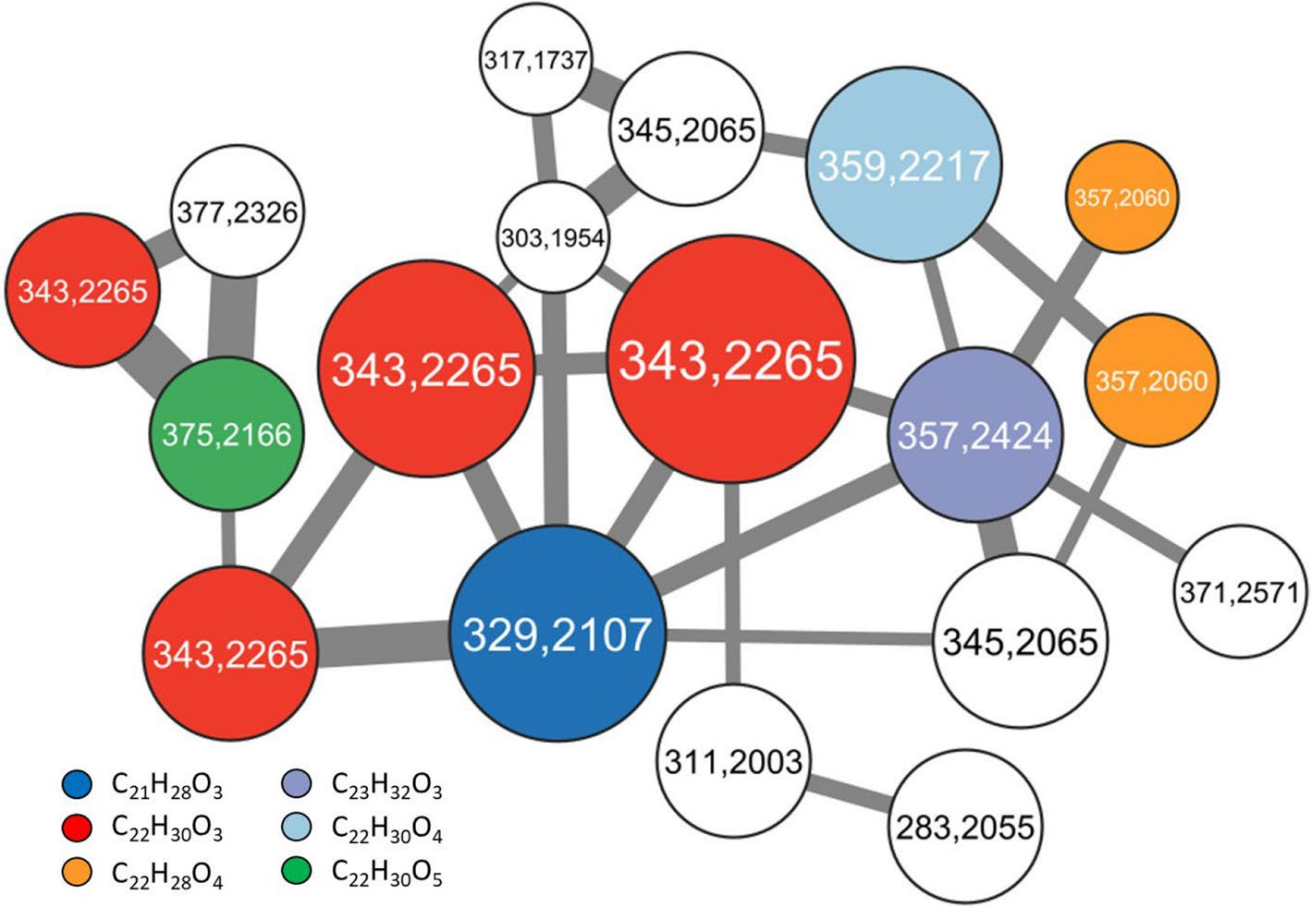
Excerpt of a molecular network showing detected polypropionates from crude extracts of *E. timida*. Putative polypropionates were clustered based on their similar MS/MS fragmentation patterns. Each node represents a polypropionate and is labelled with the detected mass. Masses that correspond to characterized polypropionates are color-coded. White nodes correspond to putative polypropionates that were not characterized yet. The node size corresponds to the production level and the edge width represents the relatedness between two compounds. The thresholds for the cluster were set to 7 minimum matched peaks and a cosine score of 0.6

To investigate the spectrum of polypropionates produced by the sea slug, three adult specimens were extracted. Analysis of high-performance liquid chromatography coupled to high resolution tandem mass spectrometry (HPLC–MS/MS) data of the crude extract resulted in the identification of putative polypropionates (Supplemental Figure S9). The obtained HPLC–MS/MS data were visualized to represent the relatedness between the putative compounds (Fig. 4). Each node in the molecular network represents a polypropionate and is labelled with the detected mass-to-charge ratio. The edge line width visualizes the degree of relatedness between two compounds. More than half of the 18 putative polypropionates detected matched characterized compounds that were reported from different *Elysia* and *Plakobranchus* species (Table 6) [108]. Additionally, eight putative polypropionates were detected that have not been structurally characterized.

**Table 6:**
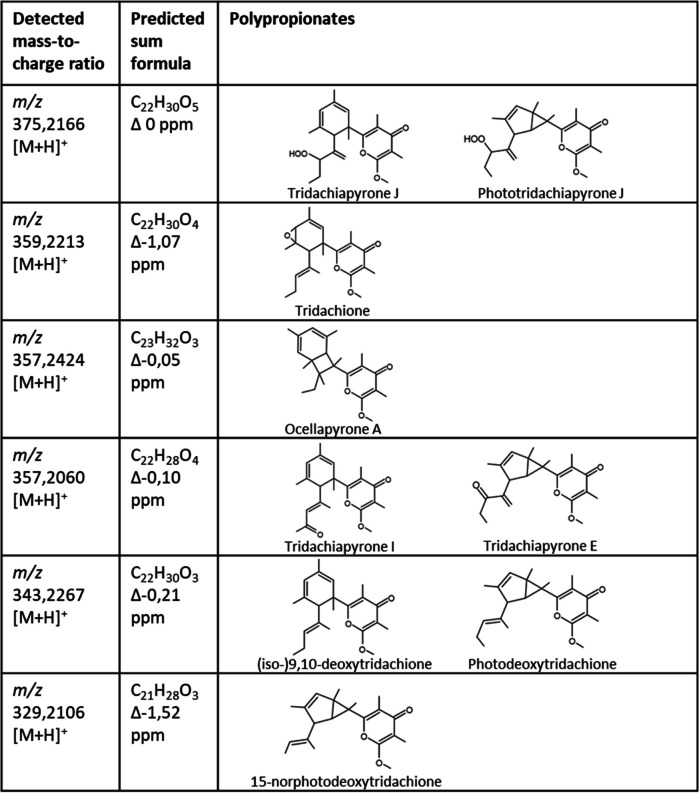
Overview of polypropionates isolated from kleptoplastic sacoglossans. Compounds were detected by LC-HR-MS/MS analysis of crude extracts. Only masses corresponding to characterized polypropionates are shown

The stereochemistry of the identified polypropionates cannot be determined by mass spectrometry. As a result, planar structures are shown. In the excerpt of the molecular network isomers of polypropionates appear as separate nodes which are labelled with the same mass-to-charge ratio. The complex polyketide scaffolds are hypothesized to derive from spontaneous light-induced cyclizations and tailoring reactions [109]. This results in the wide spectrum of polypropionates produced by sacoglossans (Supplemental Figures S9 and S10) [108].

## Discussion

The adaptive potential and remarkable survival mechanisms of Sacoglossa have been the subject of many studies. The inclusion of fully annotated Sacoglossa genomes in these studies is essential to properly investigate the genetic processes underlying functional kleptoplasty and to understand its functional role. Our *E. timida* genome assembly achieves the highest values of contiguity, of BUSCOs completeness and accuracy compared to available sacoglossan genome assemblies and therefore makes a valuable contribution to this understanding. In addition, we identified genes encoding PKS1 and PKS2 in the genome annotation of *E. timida*, suggesting that *E. timida* is a putative producer of polypropionates. It is hypothesized that polypropionates are involved in the establishment and maintenance of the association between Sacoglossa and the incorporated chloroplasts [34]. Ireland and Scheuer (1979) [110] found that in Sacoglossa, fixed carbon acquired from de novo chloroplast photosynthesis was incorporated into polypropionates. The polypropionates act via oxidative and photocyclization pathways which are suggested to behave like sunscreen and prevent the sea slugs from photosynthetic damage [110–115]. Polypropionates would therefore be needed in species with a ‘photosynthetic’ lifestyle, such as *E. timida*.

The hypothesis of HGT of algal nucleic photosynthetic genes to the nuclear DNA of the sea slug is not supported by the data presented in this study. First, despite the high coverage, all contigs assigned to Chlorophyta were assembled separately and could not be linked to other *E. timida* sequences with HiC data. Second, all blast hits returning a taxid of the two algae detected (*A. acetabulum* and *U. compressa*) are on the five contigs assigned as Chlorophyta, which implies that there are no sequences similar to these algae in the *E. timida* nuclear genome. Thirdly, only sequences similar to chloroplasts were found in the contamination screening, showing no support for the similarity to the nuclear sequences of the two detected algae sequenced together with *E. timida*. These last two statements may be less reliable, as the database probably does not reflect the true diversity of *A. acetabulum* and *U. compressa,* which would be required for a comprehensive assignment. Overall, neither the HiFi nor the HiC data support a linkage of the detected algal sequences to *E. timida* sequences, and no algal-like sequences were found in the assembly, apart from the expected chloroplast sequences (which were filtered out during contamination screening). These results are consistent with those of Maeda et al. (2021) [33], Chan et al. (2018) [32] and Wägele et al. (2011) [31], who also found no support for HGT. Nevertheless, HGT between algae and *E. timida* needs to be further investigated in future studies.

It is surprising that *U. compressa* sequences were found in addition to *A. acetabulum*. As a wild caught animal was used for sequencing, we cannot exclude the possibility of contamination from the body surface. Nevertheless, the specimen was starved in a lab culture, where *U. compressa* was not present, and previous experiments have shown that *E. timida* can feed on algae other than *A. acetabulum* [116, 117]. *Ulva compressa* is a common alga of the Catalan coast and is reported in the Global Biodiversity Information Facility (GBIF; https://www.gbif.org/es/species/52733229) for the region of Cadqués where the *E. timida* individual was sampled. Since only *U. compressa* chloroplast sequences were detected, we suggest that *U. compressa* may be a food alga for *E. timida* in the wild. This would contradict previous studies claiming that *A. acetabulum* is the only food source for *E. timida* [118, 119]*.*

Despite their high diversity, molluscs are still very poorly studied in terms of publicly accessible high-quality reference genomes, partly due to the aforementioned difficulties in DNA extraction, library preparation and sequencing [120–122]. Currently available molluscan genome assemblies in the National Center for Biotechnology Information (NCBI) cover only ~ 0.1% of the described species in the entire phylum. One reason for the often rather fragmented genome assemblies in molluscs may be that the sequencing polymerases are hindered by contaminants, such as the polysaccharide-containing mucus of molluscs, or metabolites bound to the DNA [123, 124]. The sacoglossan genome assemblies published so far are quite fragmented, with contig N50s of 0.005 to 0.45 Mb [33, 106, 107]. The reason is that in most cases long-read sequencing, but also the chromatin conformation capture library preparation did not work, as so often in molluscs. This resulted in very low sequencing yield. To enable the sequencing of these animals, we have therefore switched to the PacBio ultra-low input protocol, which includes a long-range PCR amplification step to increase the amount of DNA relative to possible contaminants and to obtain ‘artificial but clean’ DNA that can then be easily sequenced. In general, PCR amplification used in the preparation of ultra-low input libraries can lead to bias towards some genomic regions. However, using different PCR polymerases for amplification can counteract this bias and complementary amplify different genomic regions. Combining these data thus leads to improved contiguity of genome assemblies [35]. Bein et al. (2024) [35] included various species from different taxa when investigating the effect of different polymerases on long-range PCR amplification and subsequent assembly results. Other parameters, such as different sequencing machines, were not analysed by Bein et al. (2024) [35]. However, they were able to observe a different performance of KOD polymerase in mammals compared to molluscs and collembolans. Nevertheless, even in molluscs, the KOD polymerase contributes to a better contiguity of the resulting assembly when combined with data generated by PacBio’s polymerase A/B amplification. Although error rates of polymerases used for long range amplification are low (PacBio polymerases A/B: below 1 in 10^5^; KOD polymerase: 13.1 × 10^–6^), PCR errors might be present in the reads or even in the assembly. We cannot investigate the effect of these errors in this study, as no other reference for *E. timida* is known. By using the Arima HiC low-input library preparation protocol, higher cross-linking yields were achieved, ultimately resulting in increased coverage and improved distances between cross-linked genomic loci (compared to the Arima HiC standard library and Dovetail Genomic’s Omni-C library (data not shown)). With a scaffold and contig N50 of 41.8 Mb and 1.92 Mb, respectively, the *E. timida* genome assembly of this study has approximately 30-fold and fourfold better scaffold and contig N50 values than the other sacoglossan genome assemblies.

However, there is a discrepancy of total assembly length (754 Mb) and genome size estimates (FCM: 895 Mb; ModEst: 632 Mb; GenomeScope: 548 Mb). The smaller total length compared to the FCM estimate may be due to collapsed repeats that have not yet been resolved. Although PacBio sequencing was successful, the overall N50 of the HiFi reads (6 kb) may, in some cases, still be too short to resolve some long repetitive regions of the genome. The mapping-based genome size estimate of ModEst is considerably smaller than the FCM estimate and the total assembly length. ModEst assumes that differences in coverage are due to technical problems in assembly. This may not be entirely correct in our case, as differences in coverage may be caused by bias in PCR amplification. Due to its assumption, the ModEst estimate appears to be less reliable than the FCM estimate with rCV values < 5%. Similarly, genome sizes appear to be consistently underestimated by k-mer-based methods, partly due to repeats [40]. Therefore, a comparison between the total length of the high-complexity regions in the assembly and a k-mer-based estimate of genome size is useful. For example, adding the number of masked bases in the assembly (335 Mb) and the k-mer-based genome size estimate (548 Mb) yields 883 Mb, which is very close to the FCM-based genome size estimate (895 Mb). However, the fact that we performed PCR amplification prior to PacBio sequencing may have resulted in a shorter genome assembly length, and thus the mapping based genome size estimate may underestimate the true genome size, as PCR amplification can introduce errors, mainly homopolymer length changes and dinucleotide repeat compression, which may have led to this underestimation, or parts of the genome may not have been amplified at all. The number of chromosome-level sequences of 15, obtained from the HiC data for *E. timida*, is consistent with the karyotype of the sacoglossan species *Oxynoe olivacea* [125]. In earlier publications, 17 chromosomes were found in other more closely related sacoglossan species [126, 127]. However, the karyotype of *E. timida* was not included and needs to be verified in future studies.

The BUSCO values were similar to other sequenced sacoglossan species. However, the duplicated BUSCOs in the *E. timida* genome assembly were higher (4.9%) than in the other genome assemblies (< 1%). These duplications can be caused by true biological events, replicating loci which contain genes thought to be single copy orthologs. In addition, duplicated BUSCOs can result from high heterozygosity, resulting in the same genomic locus (in a diploid organism) being assembled twice, creating so-called haplotigs. Haplotypic duplications are searched for and collapsed with both hifiasm and purge_dups (here only at the ends of contigs). The heterozygosity of the *E. timida* genome (0.794%) is estimated to be higher than in other Sacoglossa (0.18%-0.42%; Supplementary Table S6; [128]). However, with only 4.9% duplicated BUSCOs, the proportion is still quite low.

Contamination can lead to major problems when dealing with genome assemblies from public databases [44, 129, 130]. Therefore, contamination screening is a fundamental part of the genome assembly. Regarding contamination, the presented assembly of *E. timida* was screened with two different tools. The advantage of blobtools over the sequence similarity-based method FCS-GX was that coverage and GC content were also taken into account. In cases where taxa are underrepresented in a database for sequence similarity searches (e.g. Mollusca), false positive and false negative hits occur more frequently. In addition, shorter sequences are less likely to be identified in general. However, taking into account the read coverage and GC content, short sequences can still be identified as contaminants with a high probability (see cluster at the bottom left of Supplemental Figure S5). False-positive hits are still possible due to the taxonomic assignment by blobtools. Nevertheless, we did not filter out sequences that were assigned to Chordata or Arthropoda because the nt database does not contain the necessary diversity of Mollusca sequences to reliably identify this phylum in a de novo assembly. The contigs of the *E. timida* assembly therefore generate hits for the closest related species in the database, likely due to conserved elements of the genome across different phyla (e.g. protein domains).

The structural annotation of the *E. timida* genome assembly shows excellent quality metrics and is the most complete in terms of BUSCOs compared to available annotated genome assemblies of Sacoglossa. In particular, the higher number of CDSs/mRNA and the longer median gene length, while total gene space is comparable, indicate a higher contiguity of annotated genes. This seems to be strongly influenced by the contiguity and accuracy of the underlying genome assembly. Furthermore, over 60% of the protein sequences resulting from the *E. timida* annotation were annotated with at least one GO term, which is 1.4- to 2.8-fold higher compared to the annotation of other Sacoglossa (Table 4). The absolute numbers of protein sequences annotated with a GO term are probably higher for other Sacoglossa due to gene fragmentation. When a gene is split between two contigs or scaffolds, it is annotated as two different genes, but if both parts are large enough to make a reliable match, a GO term (maybe even the same one) is assigned to both gene fragments. The overall functional annotation rate is lower for the other Sacoglossa, probably due to general fragmentation and sequences becoming too short to reliably match against protein sequences of known functions. However, the high number of genes annotated in other Sacoglossa may not be due to fragmentation of the assembly alone. While most of the annotation’s statistics are satisfactory, we can only speculate why EtPKS1 was not annotated by BRAKER. A gene was predicted by Augustus at the same locus, which was not taken to the final gene set of BRAKER. There are less RNAseq reads mapping to EtPKS2 (263 reads) compared to EtPKS1 (680), while both genes have a similar size (total CDS length; EtPKS1: 6785 bp; EtPKS2: 6834 bp). Therefore, the amount of RNAseq reads might not be the reason. To show that many genes could be true or false positive annotations, orthologous clustering could be performed with all available Sacoglossa and other high-quality annotations from other molluscs. As many downstream analyses (e.g. comparative, evolutionary) depend on high-quality data as input, the presented annotation will enhance or even enable future studies.

Although it has been claimed that polypropionates are not produced by the animals themselves, but by symbiotic bacteria or dietary organisms [131, 132], there is increasing evidence that animals are capable of producing various compounds themselves [133–137]. In Sacoglossa, it is assumed that the produced polypropionate pyrones contribute, among other things, to the establishment and maintenance of the association of Sacoglossa and incorporated chloroplasts [34]. The identification of genes encoding PKS1 and PKS2 in the genome annotation of *E. timida* indicates that *E. timida* is a likely producer of polypropionates. Depending on the genome annotation and the origin of the proteins, FAS, PKS1 and PKS2 genes were found in the sacoglossan assemblies. The quality of the genome assemblies and the protein sequences seemed to have a strong influence on the number of gene copies found.

Polypropionates are abundant in molluscs worldwide and have been found in the sacoglossan species *E. chlorotica*, *E. diomedea* and *P. ocellatus*. We have now expanded their presence to *E. timida*. In sacoglossans, polypropionates appear not only to bind fixed carbon from the chloroplast via de novo photosynthesis [110], but also to act via oxidative and photocyclization pathways [110–113]. These reactions may protect sacoglossans from damage caused by photosynthetic reactive oxygen products and may therefore play an important role in life with functional kleptoplasty [111–115]. Interestingly, Torres et al. (2020) [34] found the mRNA of PKSs in the transcriptome of *E. timida* [61, 114]. However, transcriptomes only show the genes that are expressed in a given tissue at a given time, resulting in a subset of all genes present in the genome. We are also aware that a blast search is not the adequate tool to do a gene orthology prediction. However, the aim in this study was getting an overview of the presence and abundance of PKS genes in the assembled and annotated sacoglossan genomes. Therefore, we chose to use the BLAST search for the PKS gene analyses. Future research might provide a deeper insight into and knowledge about the gene orthology of sacoglossan PKS genes. After the polypropionate scaffold is produced, it is decorated by tailoring enzymes. A C-methyltransferase and cytochrome P450 are probably required to produce the large number of polypropionates in Sacoglossa [108]. As in other eukaryotes, the genes for the enzymes involved in the production of natural products are not adjacent to each other. This makes it difficult to identify the corresponding genes for the decorating tailoring enzymes [34]. The genomic environments of the genes encoding PKS1 and PKS2 were searched using BLASTp, mainly yielding uncharacterised and hypothetical proteins with no indication of their catalytic activity. Only highly complete and continuous genome assemblies, such as that of *E. timida*, can provide a comprehensive picture of the genes present.

In the future, the *E. timida* genome assembly may help to shed light not only on polypropionates and their role in the functional kleptoplasty, but also on immune genes. Immune genes are well studied in cnidarians, and have recently been discussed in Sacoglossa, as the innate immune system probably plays an important role in the establishment of the process of photosymbiosis (e.g. [138–144]). Although we know more and more about the process of functional kleptoplasty, it is still unknown how especially short-term and long-term sacoglossans correctly identify the chloroplast of their food algae as a symbiont rather than a pathogen nor how chloroplasts are absorbed. Melo Clavijo et al. (2020) [144] found that sacoglossans—including *E. timida*—have a divergent collection of specific scavenger receptors and the thrombospondin-type-1 repeat protein superfamily, comparable to photosymbiotic cnidarians (e.g. [145–151]). Furthermore, they detected species-specific candidate genes that may be important for the symbiont identification in sacoglossans. We investigated the presence of polypropionate encoding genes in *E. timida* and hope that our genome assembly can also serve as a reference genome for immune gene studies in Sacoglossa. We expect the genome assembly to contribute to future genetic studies on kleptoplasty and to serve as a high-quality resource for studies on sacoglossans and molluscs in general.

## Supplementary Information

Supplementary Material 1: Supplemental Figure S1. Climate chamber in which the *E. timida* slugs were kept in artificial sea water in plastic cups as aquariums. The green tubes provided the air supply. Supplemental Table S1.Databases and tools which were used while operating InterProScan version 5.64-96.0 [101]. Supplemental Table S2.Table of PKS and fatty acid synthase (FAS) sequences from Torres et al. (2020) [34] including the animal species they were received from and the accession number. Supplemental Figure S2. Genome size estimation of *E*.* timida* using flow cytometry. The histogram shows the relative propidium iodide fluorescence intensity obtained after simultaneous analysis of *E*.* timida* 2C (in green) and the house cricket *A. domesticus* 2C as an internal standard reference (in red). The PI fluorescent dyes were excited with a solid-state laser emitting at 488 nm. The y-axis gives the counts of propidium iodide (PI) stained nuclei. The x-axis displays the relative red PI fluorescence signal. To obtain the mean relative red PI fluorescence signals, the peaks were enclosed by line segments. The percentages in brackets are the portions of all events in the histogram enclosed by the respective line segments. Supplemental Table S3. Genome size estimates from two individuals of *E. timida*. The measured individual is given in brackets. Chopping buffer was prepared as described by Galbraith et al. (1983) [152]. Propidium iodide was used as a fluorescent dye. We used the house cricket *A. domesticus* as standard reference (genome size: 2000 Mb). Supplemental Figure S3. K-mer profile and estimates based on HiFi reads. Supplemental Table S4. Sacoglossan heterozygosity values. The heterozygosity values from all species except for *E. timida*, were inferred by Theisen & Jensen (1991) [128]. Supplemental Tables S5. PacBio ultra-low library preparation based on PCR amplification with KOD Xtreme™ Hot Start DNA Polymerase (Merck). Supplemental Table S6. Sequencing output and subread mean length of the PacBio low- and ultra-input libraries. Supplemental Figure S4. HiFi read length distribution and statistics. Standard PacBio ultra-low input libraries are listed as SMRT1 and SMRT2. PacBio ultra-low libraries amplified with KOD polymerase are shown as SMRT3 (Sequel IIe) and SMRT4 (Revio). N50 values are presented in bp. Supplemental Table S7. FCS-GX contamination summary. Supplemental Table S8. FCS-GX action summary. Supplemental Figure S5. Blobplot of the assembly after polishing and purging. At this stage of the assembly process, contamination filtering with FCS was already conducted. Supplemental Table S9. Blobtools taxonomic assignment. The table shows all contigs classified as Chlorophyta by “bestsumorder”, which were filtered out among others. Sequences marked with asterisk were categorized as “HICOV” by purge_dups. Supplemental Figure S6. Blobplot of the final genome assembly. Supplemental Figure S7: Maximum likelihood phylogenetic tree of FAS, PKS1 and PKS2 transcripts from *E. timida*, *E. chlorotica*, *E. diomedea* and *P. ocellatus*. For the alignment the transcriptomic data from the sequences listed in Table 5 and Supplemental Table S2 were used. The branches are labelled with their length and scaled according to the number of substitutions per site. The percentage of trees in which the associated data clustered together is shown next to the branches. The transcript of EtPKS1 was manually constructed based on sequence homology to EcPKS1, EdPKS1 and PoPKS1 as described previously. The transcripts from *E. timida* are labelled with an asterisk. Supplemental Table S10. Number of blast hits with taxid of *Acetabularia acetabulum* or *Ulva compressa* against contigs of the polished *E. timida* genome assembly. Supplemental Table S11. Number of blast hits for targets with a taxid of *Acetabularia acetabulum* or *Ulva compressa*. All target sequences originate from a chloroplast. Supplemental Table S12. Section of the agp file from scaffolding including the 5 Chlorophyta sequences showing all resulting scaffolds containing these Chlorophyta sequences. Except for splitting one of the sequences, none was linked to other nuclear sequences of the *E. timida* genome assembly. Supplemental Figure S8. The genes encoding EtFAS, EtPKS1 and EtPKS2 are annotated in the genome of *E. timida*. The exons are labelled in black on the excerpt of the genomic sequence. The arrows present the transcript of the a) EtFAS, b) EtPKS1 and c) EtPKS2. The domains of the enzymes are presented in bubbles below the arrow. The gene encoding EtPKS1 was annotated manually based on sequence homology with the EcPKS1. The label with an x^0^ indicates an inactive domain. Supplemental Figure S9. Isotopic patterns of the putative polypropionates produced by *E. timida *and identified by HPLC-ESI-HRMS analysis. Supplemental Figure S10. HPLC-MS data of *E. timida* extracts. Base peak chromatogram (BPC) and extracted ion chromatograms (EICs) of polypropionates shown in fig. 4. The BPC shows all detected ions present in the crude extract and the EICs show the peaks corresponding to putative polypropionates. Supplemental Table S13. Result of the polypropionate blast search in the annotations of *E. timida*,*E. chlorotic*a, *E. diomedea* and *P. ocellatus*. Both unfiltered (F-) as well as filtered (F+) blast hits are shown. Supplemental Figure S11. Blobplot of the assembly after removing sequences identified as contamination and before HiC scaffolding.

## Data Availability

Raw data of PacBio HiFi reads (subreads) and raw Arima HiC reads which were used for genome assembly, raw RNAseq reads as well as the final assembly and annotation can be publicly accessed via BioProject PRJNA1119176 and this link: https://genome.senckenberg.de/download/etim/.

## Data availability

PacBio Hifi reads and Arima HiC reads which were used for genome assembly, RNAseq reads as well as the final assembly and annotation can be publicly accessed via BioProject PRJNA1119176 and this link: https://genome.senckenberg.de/download/etim/.
