# Supplementary material for: Chromosome-level genome assembly of the sacoglossan sea slug Elysia timida (Risso, 1818)
Source: BMC Genomics. 2024 Oct 7;25:941. doi: 10.1186/s12864-024-10829-7 (PMC11460185; doi:10.1186/s12864-024-10829-7)
Supplement: Supplementary file 1 — Supplementary Material 1: Supplemental Figure S1. Climate chamber in which the E. timida slugs were kept in artificial sea water in plastic cups as aquariums. The green tubes provided the air supply. Supplemental Table S1.Databases and tools which were used while operating InterProScan version 5.64-96.0 [101]. Supplemental Table S2.Table of PKS and fatty acid synthase (FAS) sequences from Torres et al. (2020) [34] including the animal species they were received from and the accession number. Supplemental Figure S2. Genome size estimation of E. timida using flow cytometry. The histogram shows the relative propidium iodide fluorescence intensity obtained after simultaneous analysis of E. timida 2C (in green) and the house cricket A. domesticus 2C as an internal standard reference (in red). The PI fluorescent dyes were excited with a solid-state laser emitting at 488 nm. The y-axis gives the counts of propidium iodide (PI) stained nuclei. The x-axis displays the relative red PI fluorescence signal. To obtain the mean relative red PI fluorescence signals, the peaks were enclosed by line segments. The percentages in brackets are the portions of all events in the histogram enclosed by the respective line segments. Supplemental Table S3. Genome size estimates from two individuals of E. timida. The measured individual is given in brackets. Chopping buffer was prepared as described by Galbraith et al. (1983) [152]. Propidium iodide was used as a fluorescent dye. We used the house cricket A. domesticus as standard reference (genome size: 2000 Mb). Supplemental Figure S3. K-mer profile and estimates based on HiFi reads. Supplemental Table S4. Sacoglossan heterozygosity values. The heterozygosity values from all species except for E. timida, were inferred by Theisen & Jensen (1991) [128]. Supplemental Tables S5. PacBio ultra-low library preparation based on PCR amplification with KOD Xtreme™ Hot Start DNA Polymerase (Merck). Supplemental Table S6. Sequencing output and subread [file 12864_2024_10829_MOESM1_ESM.docx]

**Supplements**


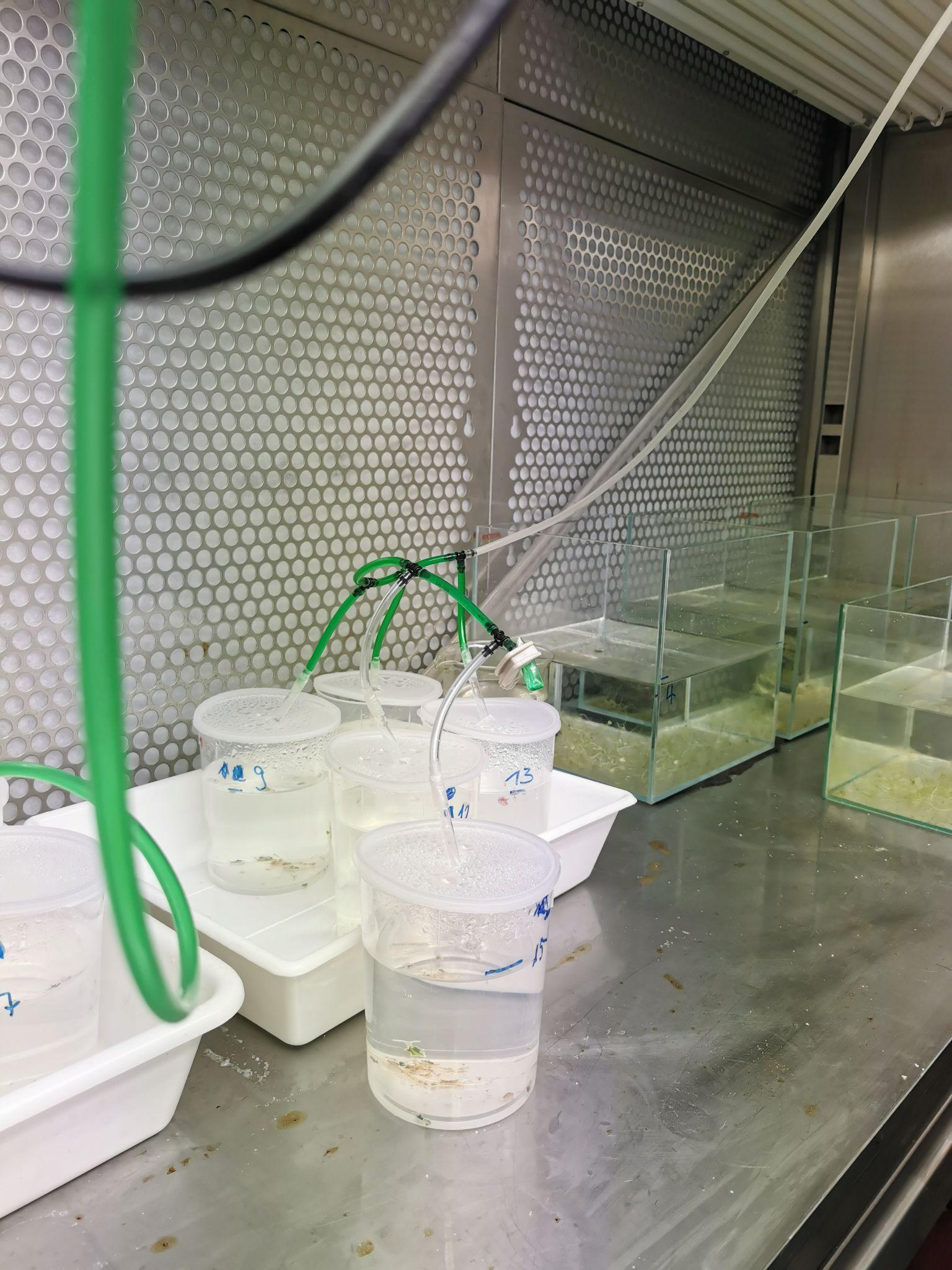


**Supplemental Figure S1.** Climate chamber in which the *E. timida* slugs were kept in artificial sea water in plastic cups as aquariums. The green tubes provided the air supply.

**Supplemental Table S1.** Databases and tools which were used while operating InterProScan version 5.64-96.0 [101].

| **Database/tool name** | **Citation** |
| --- | --- |
| AntiFam-7.0 | Eberhardt, R. Y., Haft, D. H., Punta M., Martin, M., O'Donovan, C. & Bateman, A. (2012). AntiFam: a tool to help identify spurious ORFs in protein annotation. *Database*, *2012*, bas003. |
| CDD-3.20 | Lu, S., Wang, J., Chitsaz, F., Derbyshire, M. K., Geer, R. C., Gonzales, N. R., Gwadz, M., Hurwitz, D. I., Marchler, G. H., Song, J. S., Thanki, N., Yamashita, R. A., Yang, M., Zhang, D., Zheng, C., Lanczycki, C. J. & Marchler-Bauer, A. (2020). CDD/SPARCLE: the conserved domain database in 2020. *Nucleic Acids Research*, *48*(D1), D265–D268. |
| Coils-2.2.1 | Lupas, A., Van Dyke, M. & Stock, J. (1991). Predicting Coiled Coils from Protein Sequences. *Science*, *252*, 1162-1164. |
| Gene3D-4.3.0 | Sillitoe, I., Bordin, N., Dawson, N., Waman, V. P., Ashford, P., Scholes, H. M., Pang, C. S. M., Woodridge, L., Rauer, C., Sen, N., Abbasian, M., Le Cornu, S., Datt Lam, S., Berka, K., Hutařová Varekova, I., Svobodova, R., Lees, J. & Orengo, C. A. (2021). CATH: increased structural coverage of functional space. *Nucleic Acids Research*, *49*(D1), D266–D273. |
| Hamap-2023_01 | Pedruzzi, I., Rivoire, C., Auchincloss, A. H., Coudert, E., Keller, G., de Castro, E., Baratin, D., Cuche, B. A., Bougueleret, L., Poux, S., Redaschi, N., Xenarios, I. & Bridge, A. (2015). HAMAP in 2015: updates to the protein family classification and annotation system. *Nucleic Acids Research*, *43*(D1), D1064–D1070. |
| MobiDBLite-2.0 | Piovesan, D., Necci, M., Escobedo, N., Monzon, A. M., Hatos, A., Mičetić, I., Quaglia, F., Paladin, L., Ramasamy, P., Dosztányi, Z., Vranken, W. F., Davey, N. E., Parisi, G., Fuxreiter, M. & Tosatto, S. C. E. (2021). MobiDB: intrinsically disordered proteins in 2021. *Nucleic Acids Research*, *49*(D1), D361–D367. |
| NCBIfam-12.0 | Li, W., O’Neill, K. R., Haft, D. H., DiCuccio, M., Chetvernin, V., Badretdin, A., Coulouris, G., Chitsaz, F., Derbyshire, M. K., Durkin, A. S., Gonzales, N. R., Gwadz, M., Lanczycki, C. J., Song, J. S., Thanki, N., Wang, J., Yamashita, R. A., Yang, M., Zheng, C., Marchler-Bauer, A. & Thibaud-Nissen, F. (2021). RefSeq: expanding the Prokaryotic Genome Annotation Pipeline reach with protein family model curation. *Nucleic Acids Research*, *49*(D1), D1020–D1028. |
| PANTHER-17.0 | Mi, H., Ebert, D., Muruganujan, A., Mills, C., Albou, L.-P., Mushayamaha, T. & Thomas, P. D. (2021). PANTHER version 16: a revised family classification, tree-based classification tool, enhancer regions and extensive API. *Nucleic Acids Research*, *49*(D1), D394–D403. |
| Pfam-36.0 | Mistry, J., Chuguransky, S., Williams, L., Qureshi, M., Salazar, G. A., Sonnhammer, E. L. L., Tosatto, S. C. E., Paladin, L., Raj, S., Richardson, L. J., Finn, R. D. & Bateman, A. (2021). Pfam: The protein families database in 2021. *Nucleic Acids Research*, *49*(D1), D412–D419. |
| Phobius-1.01 | Käll, L., Krogh, A. & Sonnhammer, E. L. L. (2007). Advantages of combined transmembrane topology and signal peptide prediction—the Phobius web server. *Nucleic Acids Research*, *35*(suppl_2), W429–W432. |
| PIRSF-3.10 | Nikolskaya, A. N., Arighi, C. N., Huang, H., Barker, W. C. & Wu, C. H. (2006). PIRSF Family Classification System for Protein Functional and Evolutionary Analysis. *Evolutionary Bioinformatics*, *2*, 197-209. |
| PIRSR-2021_05 | Chen, C., Wang, Q., Huang, H., Vinayaka, C. R., Garavelli, J. S., Arighi, C. N., Natale, D. A. & Wu, S. H. (2019). PIRSitePredict for protein functional site prediction using position-specific rules. *Database*, baz026. |
| PRINTS-42.0 | Attwood, T. K., Coletta, A., Muirhead, G., Pavlopoulou, A., Philippou, P. B., Popov, I., Romá-Mateo, C., Theodosiou, A. & Mitchell, A. L. (2012). The PRINTS database: a fine-grained protein sequence annotation and analysis resource—its status in 2012. *Database*, bas019. |
| ProSitePatterns-2022_05 | Sigrist, C. J. A., de Castro, E., Cerutti, L., Cuche, B. A., Hulo, N., Bridge, A., Bougueleret, L. & Xenarios, I. (2013). New and continuing developments at PROSITE. *Nucleic Acids Research*, *41*(D1), D344–D347. |
| ProSiteProfiles-2022_05 | Sigrist, C. J. A., de Castro, E., Cerutti, L., Cuche, B. A., Hulo, N., Bridge, A., Bougueleret, L. & Xenarios, I. (2013). New and continuing developments at PROSITE. *Nucleic Acids Research*, *41*(D1), D344–D347. |
| SFLD-4 | Akiva, E., Brown, S., Almonacid, D. E., Barber, A. E., Custer, A. F., Hicks, M. A., Huang, C. C., Lauck, F., Mashiyama, S. T., Meng, E. C., Mischel, D., Morris, J. H., Ojha, S., Schnoes, A. M., Stryke, D., Yunes, J. M., Ferrin, T. E., Holliday, G. L. & Babbitt, P. C. (2014). The Structure–Function Linkage Database. *Nucleic Acids Research*, *42*(D1), D521–D530. |
| SignalP_EUK-4.1 | Teufel, F., Almagro Armenteros, J. J., Johansen, A. R. Gíslason, M. H., Pihl, S. I., Tsirigos, K. D., Winther, O., Brunak, S., von Heijne, G. & Nielsen, H. (2022). SignalP 6.0 predicts all five types of signal peptides using protein language models. *Nature Biotechnology*, *40*, 1023–1025. |
| SMART-9.0 | Letunic, I., Khedkar, S. & Bork, P. (2021). SMART: recent updates, new developments and status in 2020. *Nucleic Acids Research*, *49*(D1), D458–D460. |
| SUPERFAMILY-1.75 | Pandurangan, A. P., Stahlhacke, J., Oates, M. E., Smithers, B. & Gough, J. (2019). The SUPERFAMILY 2.0 database: a significant proteome update and a new webserver. *Nucleic Acids Research*, *47*(D1), D490–D494. |
| TMHMM-2.0c | Krogh, A., Larsson, B., von Heijne, G. & Sonnhammer, E. L. (2001). Predicting transmembrane protein topology with a hidden markov model: application to complete genomes. *Journal ofMolecular Biology*, *305*(3), 567–580. |

**Supplemental Table S2.** Table of PKS and fatty acid synthase (FAS) sequences from Torres et al. (2020) [34] including the animal species they were received from and the accession number.

| **Gene name** | **Animal species** | **Accession number** |
| --- | --- | --- |
| EcFAS | *Elysia chlorotica* | MT348432 |
| EcPKS1 | *Elysia chlorotica* | MT348433 |
| EcPKS2 | *Elysia chlorotica* | MT348434 |
| EdFAS | *Elysia diomedea* | PRJNA610425 |
| EdPKS1 | *Elysia diomedea* | PRJNA610425 |
| EdPKS2 | *Elysia diomedea* | PRJNA610425 |
| PoFAS | *Plakobranchus ocellatus* | PRJNA610421 |
| PoPKS1 | *Plakobranchus ocellatus* | PRJNA610421 |
| PoPKS2 | *Plakobranchus ocellatus* | PRJNA610421 |

**
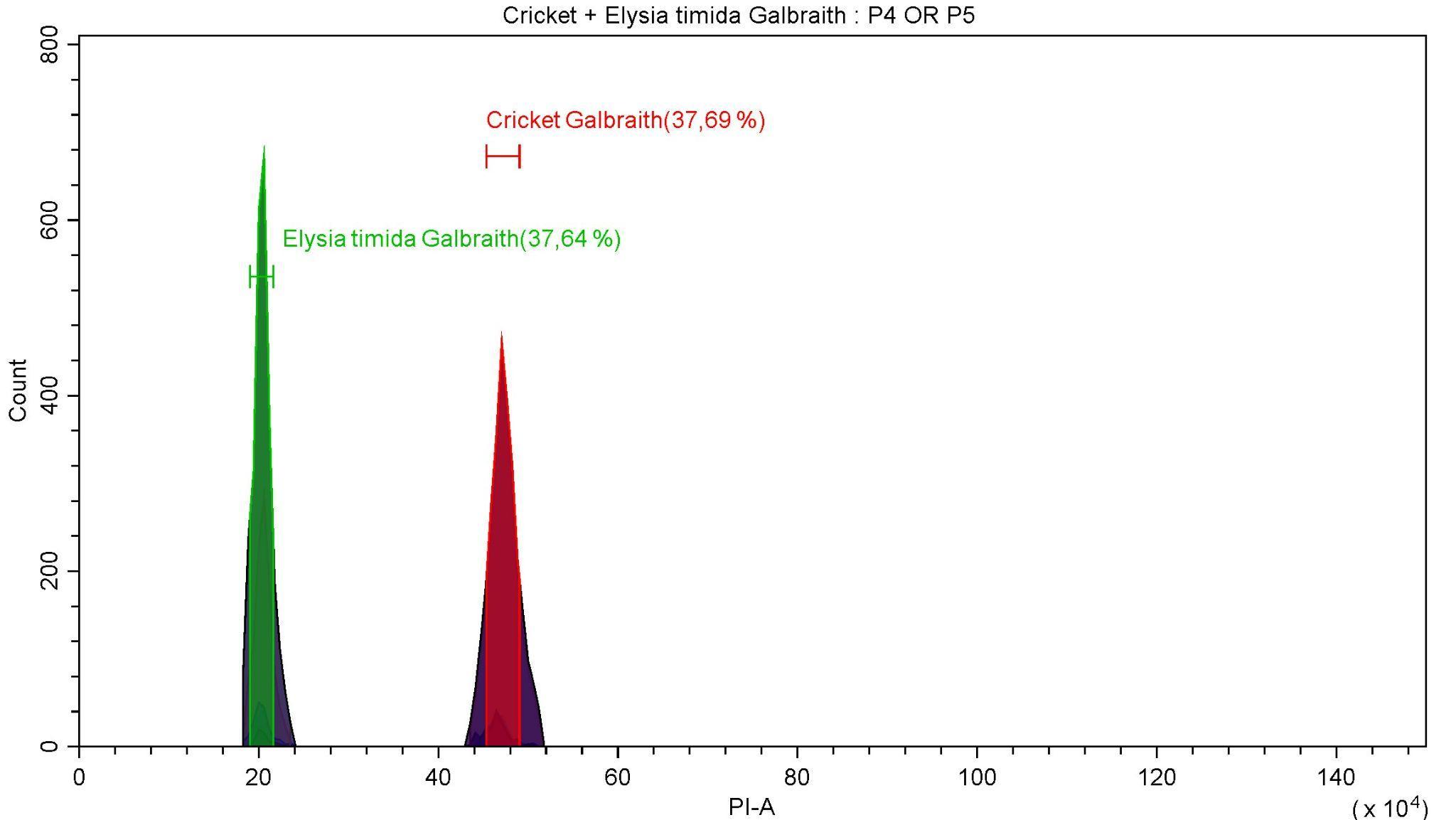
**

**Supplemental Figure S2.** Genome size estimation of *E*. *timida* using flow cytometry. The histogram shows the relative propidium iodide fluorescence intensity obtained after simultaneous analysis of *E*. *timida* 2C (in green) and the house cricket *A. domesticus* 2C as an internal standard reference (in red). The PI fluorescent dyes were excited with a solid-state laser emitting at 488 nm. The y-axis gives the counts of propidium iodide (PI) stained nuclei. The x-axis displays the relative red PI fluorescence signal. To obtain the mean relative red PI fluorescence signals, the peaks were enclosed by line segments. The percentages in brackets are the portions of all events in the histogram enclosed by the respective line segments.

**Supplemental Table S3.** Genome size estimates from two individuals of *E. timida*. The measured individual is given in brackets. Chopping buffer was prepared as described by Galbraith et al. (1983) [152]. Propidium iodide was used as a fluorescent dye. We used the house cricket *A. domesticus* as standard reference (genome size: 2000 Mb).

| **Organism (individual 1/2)** | **Genome size sample [Mb]** | **rCV value** | **Average genome size [Mb]** | |
| --- | --- | --- | --- | --- |
| *E. timida* (1) | 897.13 | 3.37% | 898.00 | 894.89 |
| *E. timida* (1) | 848.45 | 4.37% |  |  |
| *E. timida* (1) | 979.01 | 3.52% |  |  |
| *E. timida* (1) | 867.40 | 3.85% |  |  |
| *E. timida* (2) | 923.94 | 3.67% | 891.78 |  |
| *E. timida* (2) | 860.77 | 3.29% |  |  |
| *E. timida* (2) | 888.05 | 3.37% |  |  |
| *E. timida* (2) | 894.35 | 3.24% |  |  |


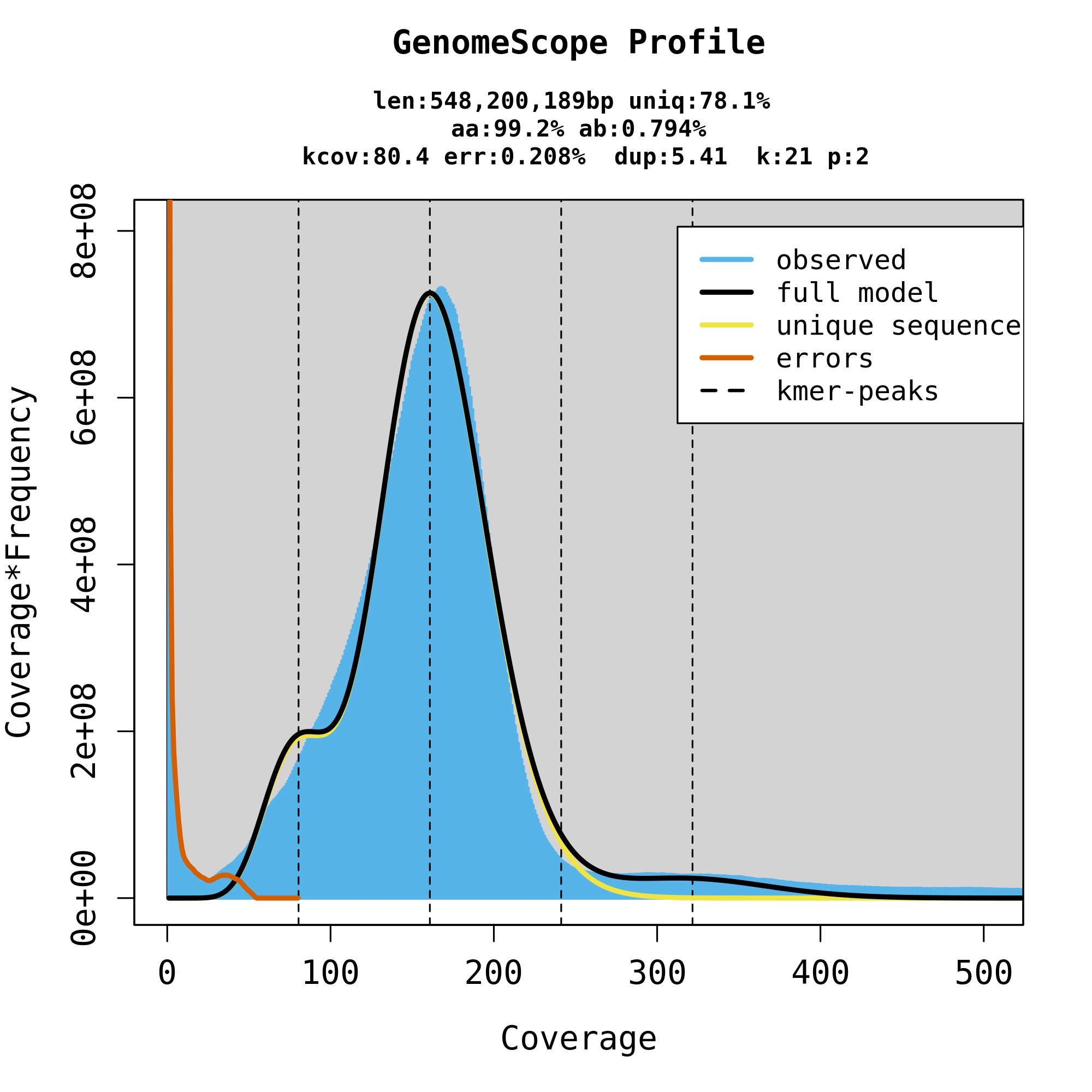
**Supplemental Figure S3.** K-mer profile and estimates based on HiFi reads.

**Supplemental Table S4.** Sacoglossan heterozygosity values. The heterozygosity values from all species except for *E. timida*, were inferred by Theisen & Jensen (1991) [128].

| **Species** | **Heterozygosity [%]** |
| --- | --- |
| *Elysia timida* | 0.794 |
| *Alderia modesta* | 0.23 |
| *Calliopaea oophaga* | 0.30 |
| *Elysia viridis* | 0.18 |
| *Ercolania nigra* | 0.42 |
| *Limapontia capitata* | 0.25 |
| *Limapontia depressa* | 0.20 |

**Supplemental Tables S5.** PacBio ultra-low library preparation based on PCR amplification with KOD Xtreme™ Hot Start DNA Polymerase (Merck)

| **Component** | **Volume (µl)** |
| --- | --- |
| 2X Xtreme buffer (in KOD kit) | 60 |
| 2 mM dNTPs (in KOD kit) | 24 |
| Ultra-low input Primer (in SMRTbell gDNA sample amplification kit) | 2.4 |
| DNA | 32 |
| KOD Polymerase (in KOD kit) | 2.4 |
| **Total** | **120.8** |

⇒ sheared gDNA input: ~ 5-6 ng

| **Cycling conditions:** | **Temperature** | **Time** | **# of cycles** |
| --- | --- | --- | --- |
| 1. Polymerase activation | 94°C | 2 min | 1 |
| 2. Denaturation | 98°C | 10 sec | 13 |
| 3. Annealing | 60°C | 30 sec |  |
| 4. Extension | 68°C | 10 min |  |
| 5. finish replication on all templates | 68°C | 5 min | 1 |
| 6. Hold at | 4 °C | ∞ |  |

**Supplemental Table S6.** Sequencing output and subread mean length of the PacBio low- and ultra-input libraries.

|  | **Low-input** | **Ultra-low input** | | **KOD amplified ultra-low input** | |
| --- | --- | --- | --- | --- | --- |
| **PacBio system** | Sequel II | Sequel IIe | Sequel IIe | Sequel IIe | Revio |
| **Sequencing mode** | CLR | CCS | CCS | CCS | CCS |
| **Polymerase read bases (Gb)** | 10 | 510 | 431 | 612 | 1,360 |


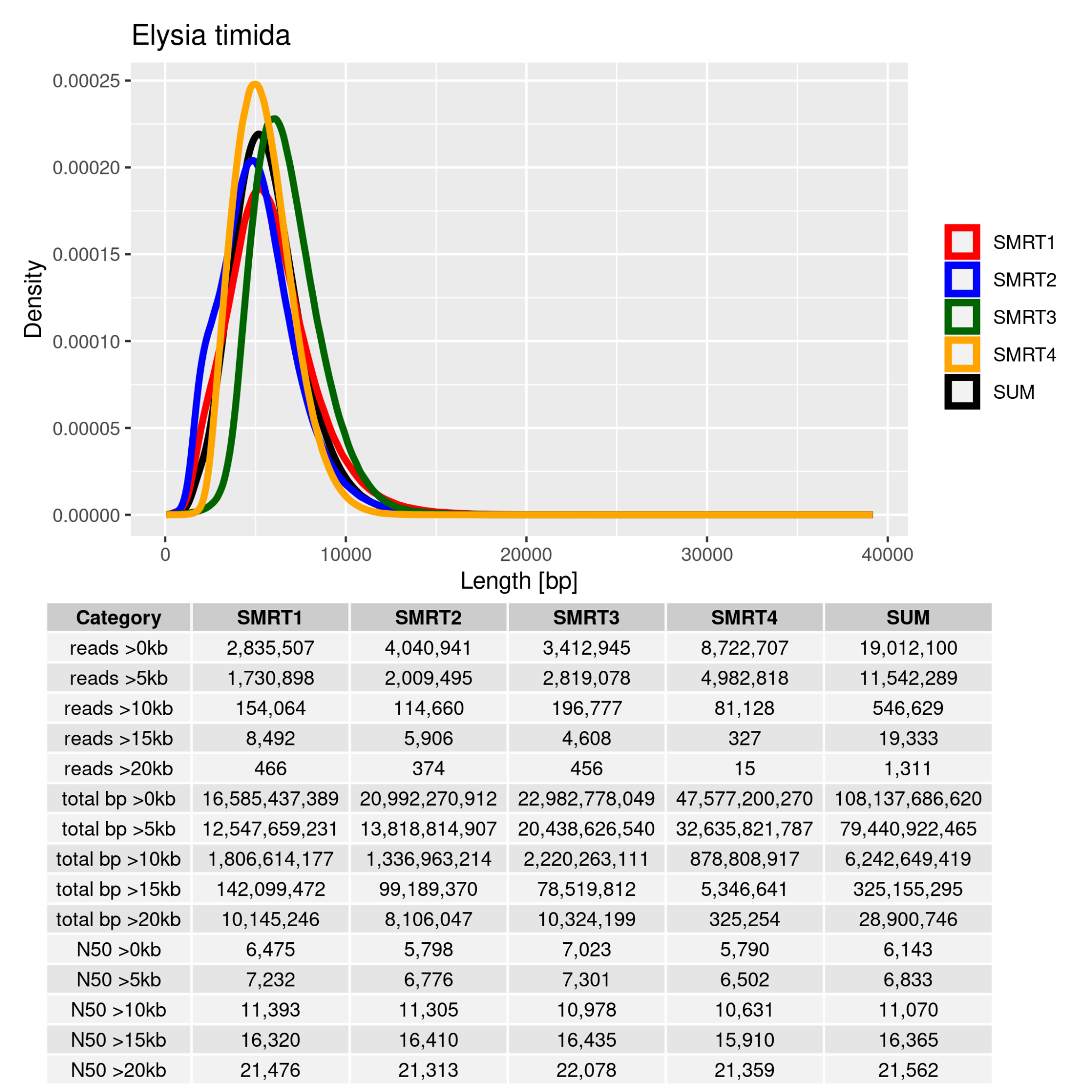


**Supplemental Figure S4.** HiFi read length distribution and statistics. Standard PacBio ultra-low input libraries are listed as SMRT1 and SMRT2. PacBio ultra-low libraries amplified with KOD polymerase are shown as SMRT3 (Sequel IIe) and SMRT4 (Revio). N50 values are presented in bp.

**Supplemental Table S7.** FCS-GX contamination summary.

|  | **seqs** | **bases** |
| --- | --- | --- |
| TOTAL | 1390 | 76016851 |
| prok:g-proteobacteria | 142 | 29973756 |
| prok:a-proteobacteria | 290 | 24308367 |
| prst:alveolates | 527 | 14776048 |
| anml:birds | 381 | 5874846 |
| prok:CFB group bacteria | 48 | 1027332 |
| prok:b-proteobacteria | 2 | 56502 |

**Supplemental Table S8.** FCS-GX action summary.

|  | **seqs** | **bases** |
| --- | --- | --- |
| TOTAL | 1390 | 76016851 |
| EXCLUDE | 871 | 53329501 |
| REVIEW | 517 | 22680217 |
| TRIM | 2 | 7133 |


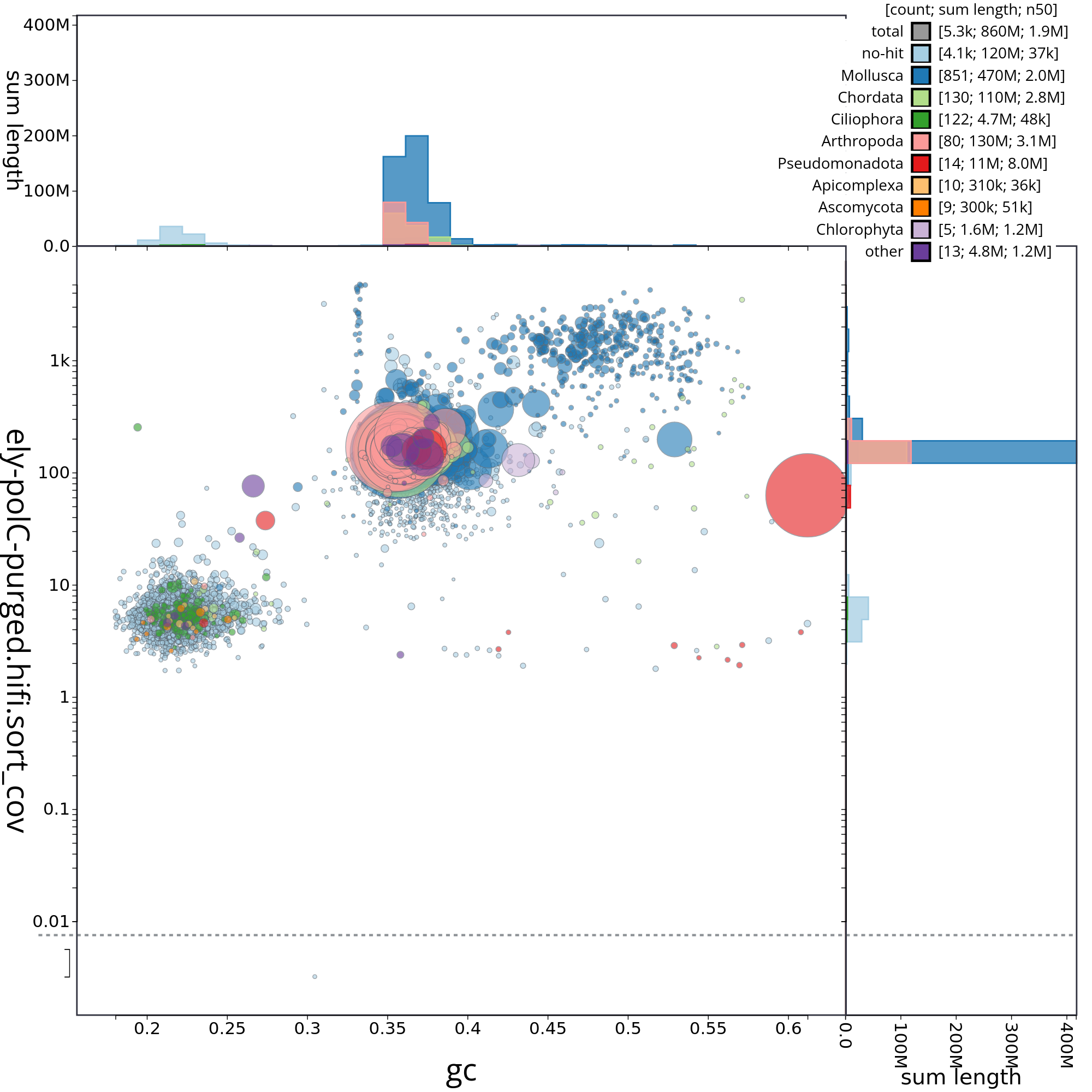


**Supplemental Figure S5.** Blobplot of the assembly after polishing and purging. At this stage of the assembly process, contamination filtering with FCS was already conducted.

**Supplemental Table S9.** Blobtools taxonomic assignment. The table shows all contigs classified as Chlorophyta by “bestsumorder”, which were filtered out among others. Sequences marked with asterisk were categorized as “HICOV” by purge_dups.

| identifiers | length | gc | ncount | HiFi coverage | bestsumorder_species |
| --- | --- | --- | --- | --- | --- |
| ptg000400l_1* | 225283 | 0.4402 | 0 | 127.447 | Acetabularia acetabulum |
| ptg000458l_1* | 1177663 | 0.4317 | 0 | 129.0502 | Acetabularia acetabulum |
| ptg004248l_1 | 179581 | 0.4115 | 0 | 84.6765 | Acetabularia acetabulum |
| ptg005766l_1 | 15908 | 0.4551 | 0 | 66.5097 | Ulva compressa |
| ptg007421l_1 | 18315 | 0.4592 | 0 | 100.5174 | Ulva compressa |


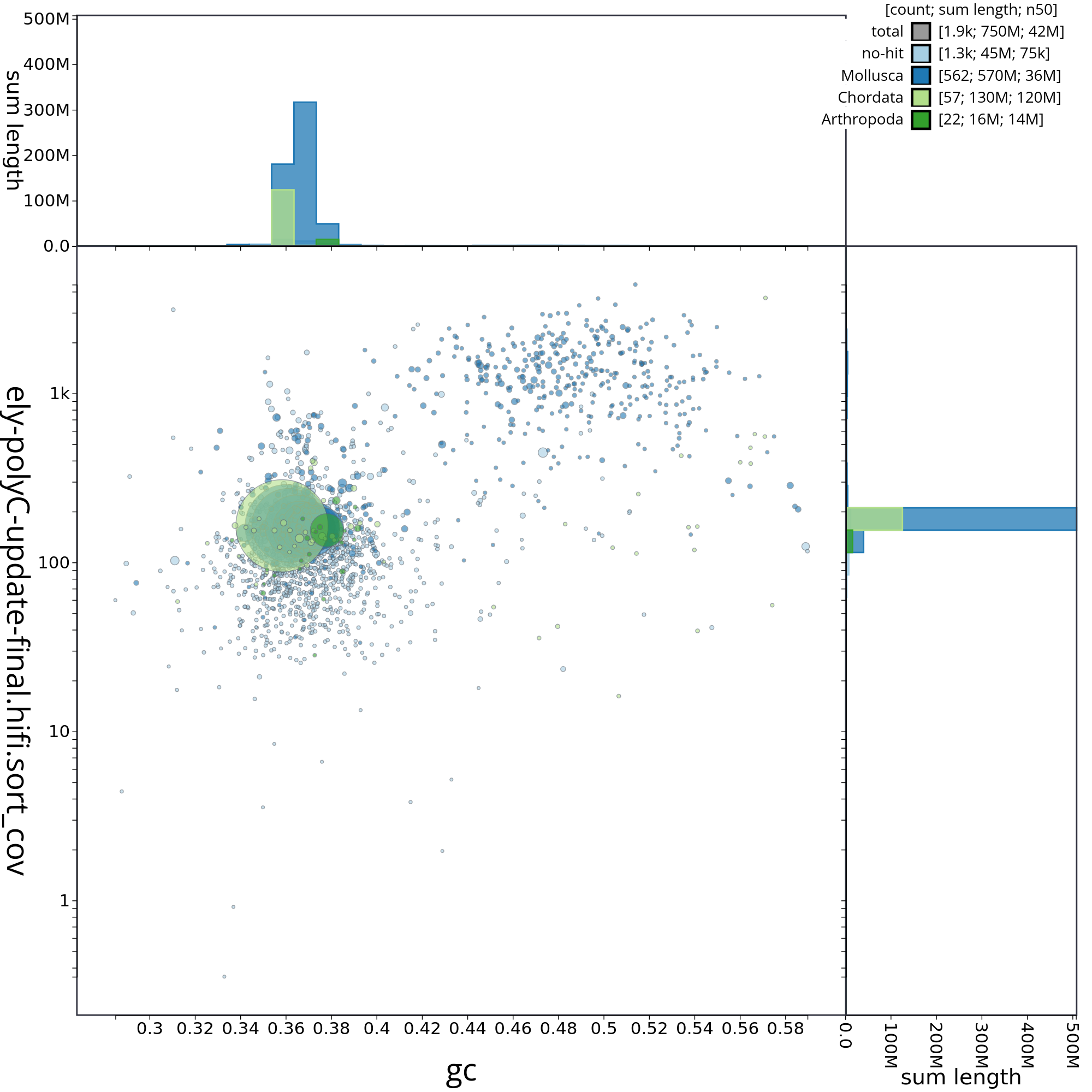


**Supplemental Figure S6.** Blobplot of the final genome assembly.


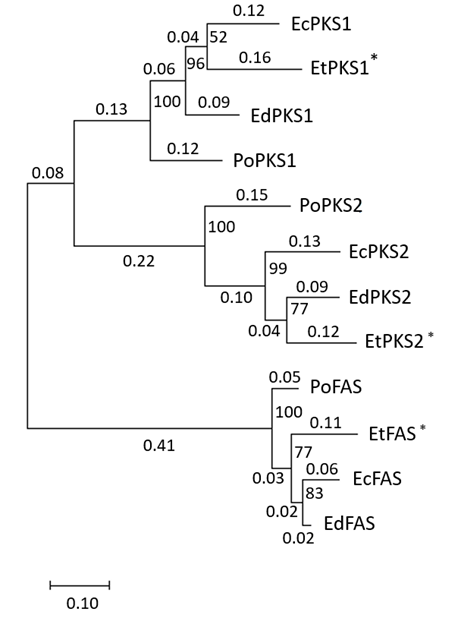


**Supplemental Figure S7:** Maximum likelihood phylogenetic tree of FAS, PKS1 and PKS2 transcripts from *E. timida*, *E. chlorotica*, *E. diomedea* and *P. ocellatus*. For the alignment the transcriptomic data from the sequences listed in Table 5 and Supplemental Table S2 were used. The branches are labelled with their length and scaled according to the number of substitutions per site. The percentage of trees in which the associated data clustered together is shown next to the branches. The transcript of EtPKS1 was manually constructed based on sequence homology to EcPKS1, EdPKS1 and PoPKS1 as described previously. The transcripts from *E. timida* are labelled with an asterisk.

**Supplemental Table S10.** Number of blast hits with taxid of *Acetabularia acetabulum* or *Ulva compressa* against contigs of the polished *E. timida* genome assembly.

|  | *Acetabularia acetabulum* | *Ulva compressa* |
| --- | --- | --- |
| ptg000400l_1 | 7 | 47 |
| ptg000458l_1 | 26 | 121 |
| ptg004248l_1 | 4 | 0 |
| ptg005766l_1 | 1 | 6 |
| ptg007421l_1 | 1 | 6 |

**Supplemental Table S11.** Number of blast hits for targets with a taxid of *Acetabularia acetabulum* or *Ulva compressa*. All target sequences originate from a chloroplast.

| Number of blast hits | Accession number | Definition |
| --- | --- | --- |
| 30 | NC_050739.1 | Ulva compressa chloroplast, complete genome. |
| 30 | MW548841.1 | Ulva compressa voucher UNA00072687 chloroplast, complete genome. |
| 30 | MW353781.1 | Ulva compressa isolate Uco3 plastid, complete genome. |
| 30 | MT916929.1 | Ulva compressa chloroplast, complete genome. |
| 30 | MK069585.1 | Ulva compressa chloroplast, partial genome. |
| 29 | MW344287.1 | Ulva compressa isolate Uco2 plastid, complete genome. |
| 5 | HG794360.1 | Acetabularia acetabulum chloroplast mRNA for photosystem II protein D1 (psbA gene), strain DI1. |
| 4 | HG518444.1 | Acetabularia acetabulum chlorplast psaB gene for PSI P700  apoprotein A2, strain DI1. |
| 3 | HG518426.1 | Acetabularia acetabulum chlorplast atpA gene for F1 sector of membrane-bound ATP synthase, alpha subunit, strain DI1. |
| 3 | AB012085.1 | Acetabularia acetabulum mRNA for Cl--pumping ATPase a subunit, complete cds. |
| 2 | X05806.1 | Acetabularia mediterranea chloroplast ORF DNA homologous to Drosophila per gene. |
| 1 | HG518471.1 | Acetabularia acetabulum chlorplast tufA gene for elongation factor 1-alpha, strain DI1. |
| 1 | HG518467.1 | Acetabularia acetabulum chlorplast rps4 gene for chloroplast 30S ribosomal protein S4, strain DI1. |
| 1 | HG518459.1 | Acetabularia acetabulum chlorplast rpl2 gene for chloroplast 50S ribosomal protein L2, strain DI1. |
| 1 | HG518454.1 | Acetabularia acetabulum chlorplast rbcL gene for RuBisCO large subunit, strain DI1. |
| 1 | HG518447.1 | Acetabularia acetabulum chlorplast psbB gene for photosystem II P680 chlorophyll A apoprotein, strain DI1. |
| 1 | HG518445.1 | Acetabularia acetabulum chlorplast psaC gene for photosystem I iron-sulfur center, strain DI1. |
| 1 | HG518441.1 | Acetabularia acetabulum chlorplast petB gene for cytochrome b6, strain DI1. |
| 1 | HG518440.1 | Acetabularia acetabulum chlorplast petA gene for apocytochrome f precursor, strain DI1. |
| 1 | HG518438.1 | Acetabularia acetabulum chlorplast ftsH gene for cell division protein FtsH-like protease, strain DI1. |
| 1 | HG518437.1 | Acetabularia acetabulum chlorplast partial cysT gene for sulfate transport protein, strain DI1. |
| 1 | HG518436.1 | Acetabularia acetabulum chlorplast cysA gene for sulfate ABC transporter protein, strain DI1. |
| 1 | HG518435.1 | Acetabularia acetabulum chlorplast clpP gene for ATP-dependent Clp protease proteolytic subunit, strain DI1. |
| 1 | HG518434.1 | Acetabularia acetabulum chlorplast chlN gene for light-independent protochlorophyllide reductase subunit N, strain DI1. |
| 1 | HG518433.1 | Acetabularia acetabulum chlorplast chlL gene for light-independent protochlorophyllide reductase iron-sulfer ATP-binding protein, strain DI1. |
| 1 | HG518432.1 | Acetabularia acetabulum chlorplast chlI gene for magnesium-chelatase subunit chlI, strain DI1. |
| 1 | HG518431.1 | Acetabularia acetabulum chlorplast chlB gene for light-independent protochlorophyllide reductase subunit B, strain DI1. |
| 1 | HG518427.1 | Acetabularia acetabulum chlorplast atpB gene for F0 sector of membrane-bound ATP synthase, subunit a, strain DI1. |
| 1 | HG518425.1 | Acetabularia acetabulum chlorplast accD gene for acetyl-CoA carboxylase beta-carboxyl transferase subunit, strain DI1. |
| 1 | FJ715714.1 | Acetabularia acetabulum strain LB 2694 ribulose-1,5-bisphosphate carboxylase/oxygenase large subunit (rbcL) gene, partial cds; chloroplast. |
| 1 | FJ535854.1 | Acetabularia acetabulum elongation factor Tu (tufA) gene, partial cds; chloroplast. |
| 1 | D85078.1 | Acetabularia acetabulum mRNA for adenosine triphosphatase, partial cds. |
| 1 | AY177738.1 | Acetabularia acetabulum ribulose-1,5-bisphosphate carboxylase/oxygenase large subunit (rbcL) gene, partial cds; chloroplast gene for chloroplast product. |
| 1 | AF387106.1 | Enteromorpha compressa ribulose bisphosphate carboxylase large subunit (rbcL) gene, partial cds; chloroplast gene for chloroplast product. |

**Supplemental Table S12.** Section of the agp file from scaffolding including the 5 Chlorophyta sequences showing all resulting scaffolds containing these Chlorophyta sequences. Except for splitting one of the sequences, none was linked to other nuclear sequences of the *E. timida* genome assembly.

| ctg00000579.1 | 1 | 1177663 | 1 | W | ptg000458l_1 | 1 | 1177663 | + |
| --- | --- | --- | --- | --- | --- | --- | --- | --- |
| ctg00000580.1 | 1 | 225283 | 1 | W | ptg000400l_1 | 1 | 225283 | + |
| ctg00000684.1 | 1 | 132581 | 1 | W | ptg004248l_1 | 47001 | 179581 | + |
| ctg00000685.1 | 1 | 47000 | 1 | W | ptg004248l_1 | 1 | 47000 | + |
| ctg00001465.1 | 1 | 18315 | 1 | W | ptg007421l_1 | 1 | 18315 | + |
| ctg00001589.1 | 1 | 15908 | 1 | W | ptg005766l_1 | 1 | 15908 | + |


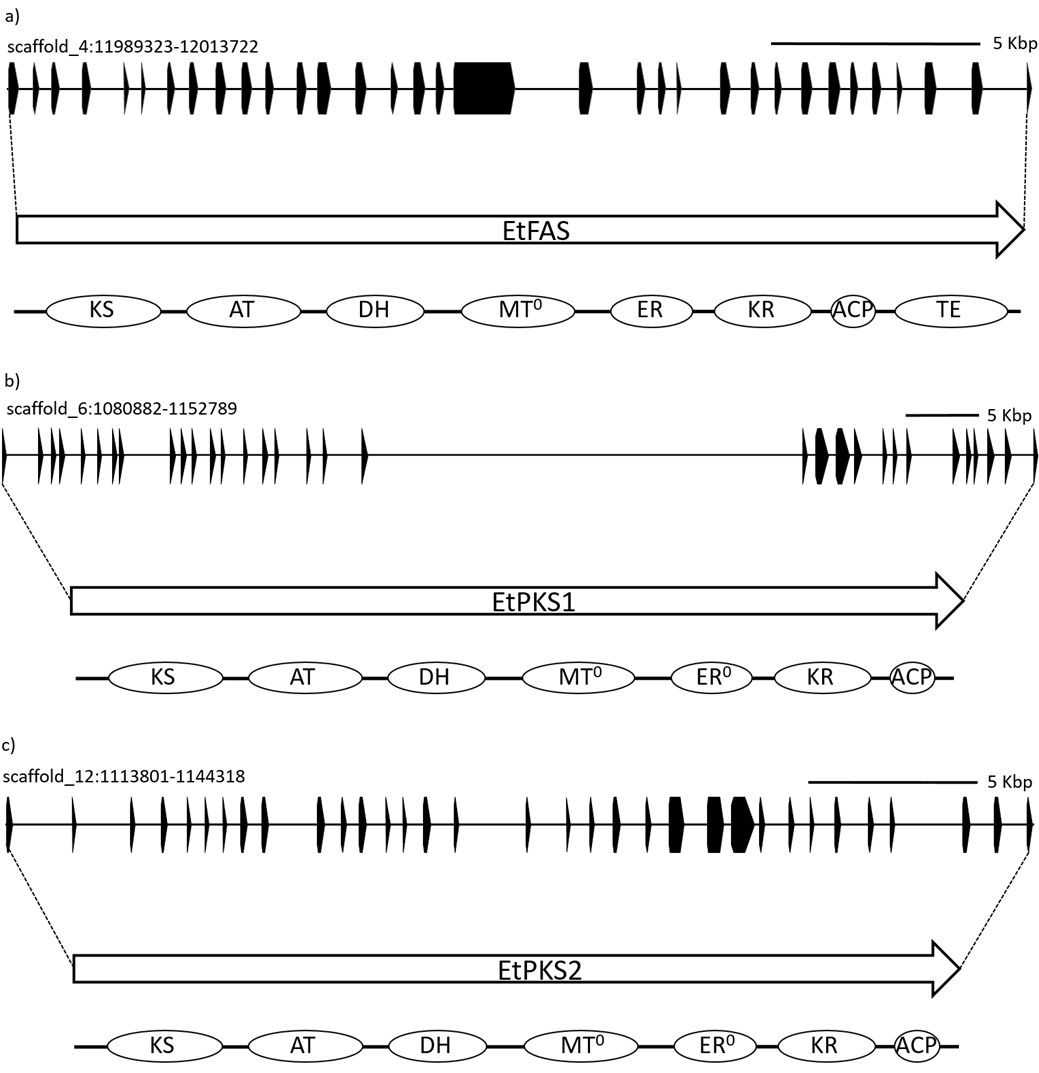


**Supplemental Figure S8.** The genes encoding EtFAS, EtPKS1 and EtPKS2 are annotated in the genome of *E. timida*. The exons are labelled in black on the excerpt of the genomic sequence. The arrows present the transcript of the a) EtFAS, b) EtPKS1 and c) EtPKS2. The domains of the enzymes are presented in bubbles below the arrow. The gene encoding EtPKS1 was annotated manually based on sequence homology with the EcPKS1. The label with an x^0^ indicates an inactive domain.


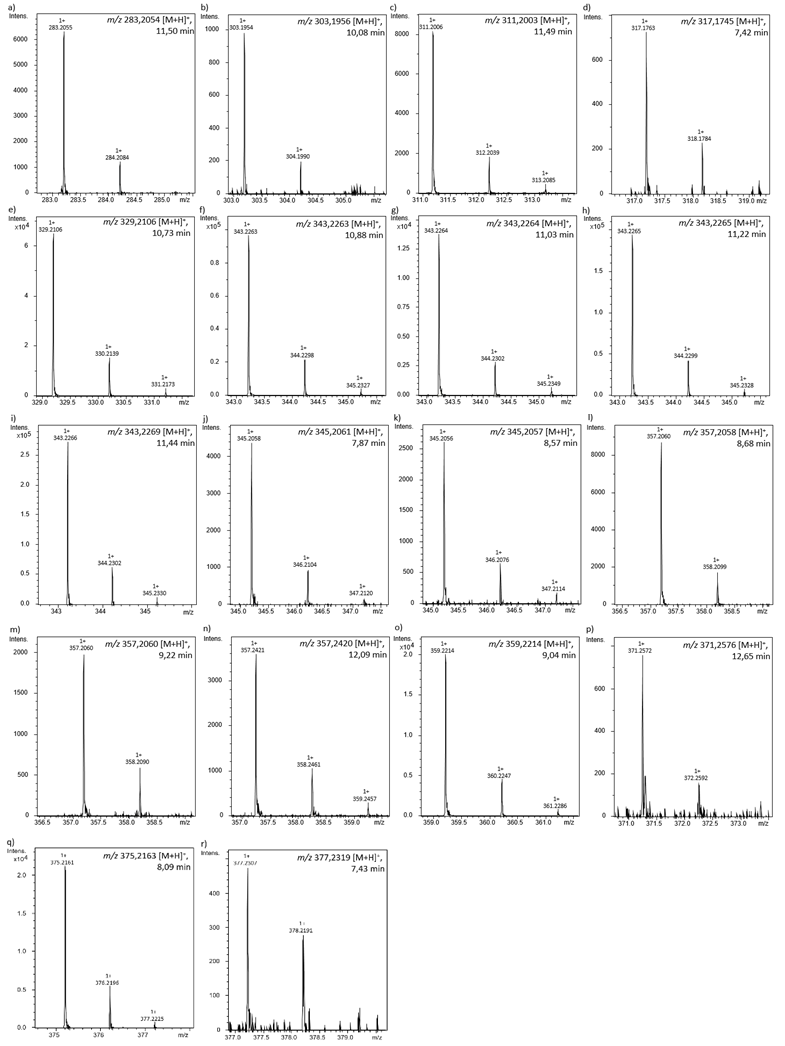


**Supplemental Figure S9.** Isotopic patterns of the putative polypropionates produced by *E. timida* and identified by HPLC-ESI-HRMS analysis.


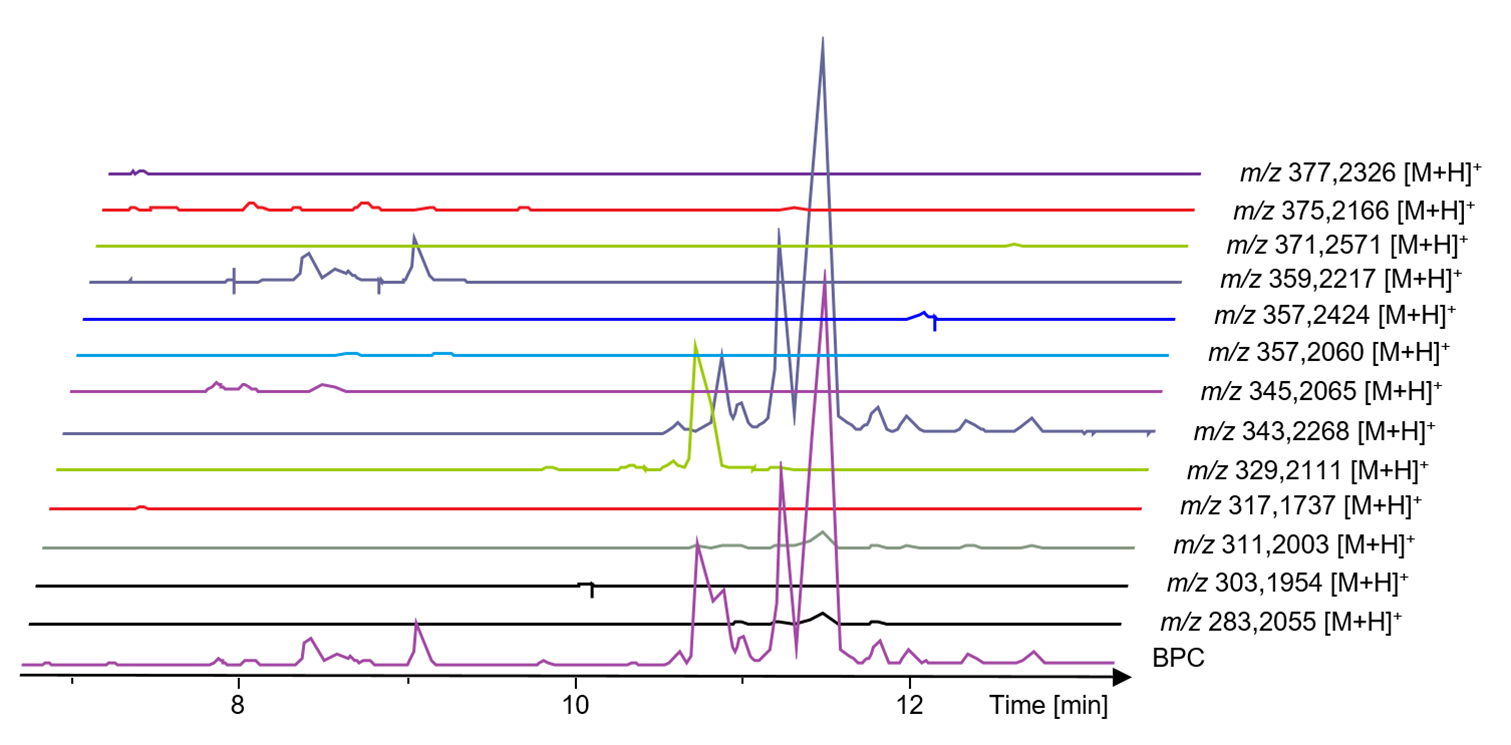


**Supplemental Figure S10.** HPLC-MS data of *E. timida* extracts. Base peak chromatogram (BPC) and extracted ion chromatograms (EICs) of polypropionates shown in figure 4. The BPC shows all detected ions present in the crude extract and the EICs show the peaks corresponding to putative polypropionates.

**Supplemental Table S13.** Result of the polypropionate blast search in the annotations of *E. timida*, *E. chlorotic*a, *E. diomedea* and *P. ocellatus*. Both unfiltered (F-) as well as filtered (F+) blast hits are shown.

|  | ***E. chlorotica polypropionates*** | | | ***E. diomedea polypropionates*** | | | ***P. ocellatus polypropionates*** | | |
| --- | --- | --- | --- | --- | --- | --- | --- | --- | --- |
|  | FAS | PKS1 | PKS2 | Edfas | Edpks1 | Edpks2 | Pofas | Popks1 | Popks2 |
| *E. timida* annotation (F-) | 32 | 34 | 36 | 7 | 10 | 10 | 7 | 8 | 12 |
| *E. timida* annotation with filter (F+) | 1 | 0 | 0 | 1 | 0 | 1 | 0 | 0 | 0 |
| *E. chlorotica* annotation (F-) | 33 | 40 | 33 | 15 | 15 | 22 | 13 | 16 | 18 |
| *E. chlorotica* annotation (F+) | 1 | 1 | 3 | 1 | 0 | 2 | 1 | 0 | 0 |
| *E. crispata* annotation (F-) | 49 | 46 | 41 | 22 | 14 | 17 | 22 | 18 | 19 |
| *E. crispata* annotation (F+) | 1 | 1 | 1 | 1 | 1 | 3 | 1 | 0 | 0 |
| *E. marginata* annotation (F-) | 73 | 71 | 76 | 24 | 19 | 26 | 30 | 23 | 22 |
| *E. marginata* annotation (F+) | 2 | 4 | 2 | 3 | 1 | 2 | 2 | 1 | 0 |
| *P. ocellatus* annotation (F-) | 46 | 44 | 49 | 20 | 15 | 17 | 20 | 15 | 12 |
| *P. ocellatus* annotation (F) | 0 | 0 | 0 | 1 | 1 | 0 | 1 | 1 | 2 |


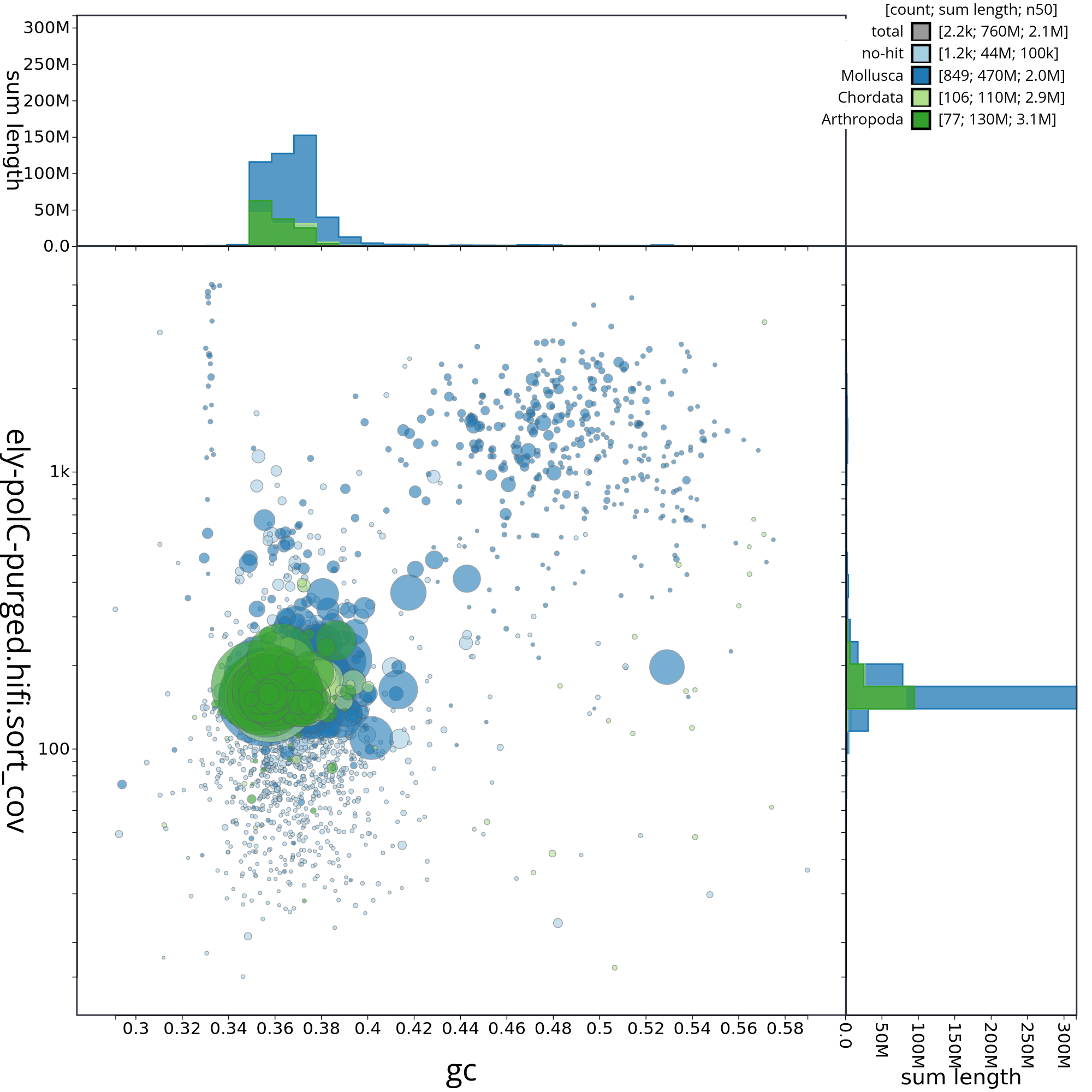


**Supplemental Figure S11.** Blobplot of the assembly after removing sequences identified as contamination and before H
